# Soil and genotype-driven resource allocation trade-offs shape root exudation across crop species

**DOI:** 10.1007/s11104-026-08721-2

**Published:** 2026-06-18

**Authors:** Henning Schwalm, Carmen Escudero-Martinez, Molly Brown, Lawrie Brown, David Roberts, Susan M. Mitchell, Ignacio Romero Lozano, Natacha Bodenhausen, Davide Bulgarelli, Kelly Houston, Timothy S. George, Eva Oburger

**Affiliations:** 1 Institute of Soil Research, Department of Ecosystem Management, Climate and Biodiversity, BOKU University, Tulln an der Donau, Austria; 2 Plant Sciences, School of Life Sciences, University of Dundee, Dundee, UK; 3 Plant-Microorganism Interaction Department, Institute of Natural Resources and Agrobiology of Salamanca, Consejo Superior de Investigaciones Científicas (IRNASA-CSIC), Salamanca, Spain; 4 The James Hutton Institute, Invergowrie, Dundee, UK; 5 Department of Soil Sciences, FiBL Switzerland, Frick, Switzerland

**Keywords:** Carbon partitioning, Rhizosphere processes, Root economic space framework, Root morphology

## Abstract

**Aims:**

Root exudates play a central role in rhizosphere processes, many of which support plant growth. While increased exudation under abiotic stresses has been frequently linked to enhanced plant resilience, crop- and genotype- and soil-specific exudation patterns under non-stress conditions remain poorly understood. This study aimed to assess how soil type and genotype influence root exudation in major and emerging European crops and to explore how root morphology and plant growth are related to exudation.

**Methods:**

Four genotypes each of barley (*Hordeum vulgare*), faba bean (*Vicia faba*), potato (*Solanum tuberosum*), and sweet potato (*Ipomoea batatas* (L.) Lam.) were grown in three distinct European soils under non-stress conditions. Exudates were collected using a soil-hydroponic-hybrid approach and analysed for dissolved organic carbon and nitrogen, as well as total carbohydrates, amino acids, and phenolic compounds. Biomass and root morphology were assessed to examine correlations with exudation patterns.

**Results:**

Results showed that soil type and genotype affected exudation patterns, but their influence varied by crop. Plant growth was negatively correlated with exudation rates across most crops, likely reflecting a trade-off in carbon and nitrogen allocation between biomass accumulation and rhizodeposition. Root morphological traits partly correlated with root exudation rates, but no universal relationships were detected across crops.

**Conclusion:**

Our results provide novel insights into belowground resource partitioning and broaden the understanding of exudation patterns to previously underexplored crops, highlighting resource allocation trade-offs shaped by genotype and soil as important drivers of exudation dynamics.

**Supplementary Information:**

The online version contains supplementary material available at 10.1007/s11104-026-08721-2.

## Introduction

The future sustainability of agriculture faces multiple challenges, including growing food demand, a decline in fertile land, and the destabilizing impacts of climate change (Blum 2013; Vigani et al. 2024). Addressing these agricultural issues requires large-scale innovations, for example, the cultivation of drought-tolerant tropical crops like sweet potato (*Ipomoea batatas* (L.) Lam.) in northern latitudes (Kännaste et al. 2023; Sapakhova et al. 2023), but also small-scale strategies that focus on optimising the use of root and rhizosphere traits to enhance plant resilience (Oburger et al. 2022). One such trait gaining increasing attention is root exudation, a key process through which plants interact with their immediate soil environment. Root exudates are plant-derived organic compounds released by living roots into the surrounding rhizosphere, where they drive feedback loops among plants, soil, and microbial communities, shaping rhizosphere processes (Oburger and Jones 2018). These exudate-driven rhizosphere processes play important roles in plant function and can enhance resilience against various abiotic stresses (Chai and Schachtman 2022; Ober et al. 2021). For example, under nutrient deficiency, increased exudation rates of mobilising metabolites can improve plant nutrient acquisition (Hoffland et al. 2006). Likewise, exuded mucilage can reinforce the soil hydraulic conductivity, enhancing plant water uptake under drought (Carminati et al. 2016). Such studies emphasise a positive role of pronounced exudation in plant functioning under abiotic stresses and suggest that enhanced exudation may also correlate with improved growth under non-stress conditions. However, carbon (C) and nitrogen (N) trade-offs between exudation benefits and biomass production may differ when stress is absent. Whether increased exudation also supports plant growth in non-stress environments (defined here as the absence of nutrient, water, light, and temperature limitations) remains unclear.

This question is particularly relevant in European agricultural soils, which often receive excessive fertilizer inputs, thereby minimising nutrient deficiency as a stress factor in crop production (Leip et al. 2011; Panagos et al. 2022). Even under fertilized conditions, several studies have demonstrated distinct exudation profiles of crops grown in different agricultural soil types (Neumann et al. 2014; Maurer et al. 2021; Santangeli et al. 2024). Soil types vary in texture, aeration, water retention, nutrient availability, as well as microbial communities, all of which shape nutrient dynamics in the rhizosphere. Root exudation is closely linked to these dynamics because it is expected to be partly driven by diffusion along a concentration gradient between root tissues and the surrounding soil: small soil metabolite concentrations are thought to stimulate exudation, whereas large concentrations may suppress it (Canarini et al. 2019). In C-rich soils, elevated microbial activity is likely to rapidly metabolise root-exuded primary metabolites such as sugars and amino acids, thereby maintaining steep concentration gradients and sustaining exudation (Canarini et al. 2019). Also, soil texture and mineralogy influence soil nutrient dynamics and thus root exudation rates. For example, maize (*Zea mays*) showed contrasting exudation rates per root surface area after growth in loamy versus sandy soil, releasing more C after growth in the loamy soil (Santangeli et al. 2024). Thus, different agricultural soil types can affect root exudation, but it remains unclear to what extent such soil-specific exudation dynamics are transferable to other crop species like barley, faba bean, potato, or sweet potato. In addition, it remains challenging to disentangle the mechanisms driving soil-specific exudation patterns, as a full mechanistic understanding of root exudation drivers in both in situ (soil-grown) and ex situ (short-term hydroponic sampling after soil growth) contexts is still lacking.

Genotypic variation within crop species is recognized as another driver of root and rhizosphere traits. Differences in exudate composition have been linked to genetic variation in wheat (*Triticum turgidum* L.) (Iannucci et al. 2017), maize (Lopez-Guerrero et al. 2022), and *Arabidopsis thaliana* (Mönchgesang et al. 2016). However, the strength of the relationship between plant genetics and root exudation profiles remains poorly understood, and it is unclear whether subtle genetic differences among genetically similar commercial elite genotypes drive meaningful variation, a knowledge gap this study addresses.

Root exudates are deeply embedded in rhizosphere processes and rapidly interact with soil and microbes, making it difficult to sample them without altering their composition (Oburger and Jones 2018). This methodological challenge motivated us to seek reliable proxies for exudation. Given the strong links between exudates and plant traits, root morphological traits may offer such proxies (Rathore et al. 2023; von Haden et al. 2024). Core morphological traits such as specific root length (SRL), root diameter (RD), and root tissue density (RTD) are organized within the Root Economic Space (RES) framework to describe root resource investment strategies (Bergmann et al. 2020; Matthus et al. 2025). These traits covary along two independent gradients: the conservation gradient ranges from tissue metabolic activity to tissue conservation and is defined by a negative correlation between root nitrogen (a proxy for metabolic activity) and RTD (a proxy for structural investment). The collaboration gradient ranges from self-reliant resource acquisition to mycorrhizal dependence and is defined by a negative correlation between SRL (a proxy for self-reliance) and RD (a proxy for resource acquisition outsourced to mycorrhizal partners). Recent studies have proposed extending this framework to include root exudation, revealing correlations between exudation and RES traits (Kumar et al. 2024; Sun et al. 2021; von Haden et al. 2024; Wen et al. 2022). These findings suggest that RES morphological traits may serve as practical proxies for studying root exudation. For example, higher RTD is often a predictor of slow-growing roots and low C exudation rates (Kumar et al. 2024; Sun et al. 2021). However, correlations between exudation and other morphological traits, such as RD and SRL, were inconsistent across studies (Jiang et al. 2022; Santangeli et al. 2024; Sun et al. 2021; von Haden et al. 2024; Wang et al. 2021; Williams et al. 2022; Yin et al. 2023), likely reflecting variation in growth conditions and sampling methodologies. To reduce methodological variability, we applied a standardized sampling approach across crop species to test exudation-RES relationships and to identify reliable root morphological proxies for exudation.

Ultimately, this work aimed to expand our understanding of soil and genotype-specific exudation in established crops (barley, faba bean, potato) and an emerging crop (sweet potato) grown under non-stress conditions. We quantified total C and N inputs as well as the exudation of carbohydrates, amino acids, and phenolics. We hypothesized that (H1) irrespective of the crop species investigated, root exudation rates differ between agronomically relevant genotypes and soil types, (H2) exudation rates positively correlate with plant biomass production under non-stress conditions, and (H3) root morphological traits can serve as proxies for exudation rates.

## Materials and methods

### Plant material and experimental design

Four independent pot experiments with different crop species (i.e., barley (*Hordeum vulgare*), faba bean (*Vicia faba*), potato (*Solanum tuberosum*), and sweet potato (*Ipomoea batatas* (L.) Lam.) were conducted under controlled environmental conditions. Four genotypes of each crop (Barley: Fairytale, KWS Irina, Laureate, RGT Planet; Faba bean: Ascot, ILB1814, Lynx, Zoran; Potato: Cara, Desiree, Innovator, Duke of York; Sweet potato: Boniato, Blesbok, Minamiyutaka, Ivis White) were grown in three different soil types. The genotypes were selected from a pool of elite lines provided by the Roots2Resilience project. The selection aimed to capture contrasting phenotypes. However, the choice of lines was constrained by the availability of plant material (seeds, tubers, cuttings) for both controlled-environment and large-scale field experiments conducted across multiple project partners. Consequently, several phenotypically similar varieties were included. Genetic similarity among barley genotypes was obtained from in-house data, which are also available in Schreiber et al. (2024), using the 50 K Illumina Infinium iSelect SNP array (Bayer et al. 2017). For potato, the genotyping data was obtained from Sharma et al. (2024), who used the Infinium 8 K Potato SNP Array (Felcher et al. 2012; Hamilton et al. 2011). Pairwise similarity was calculated using the allelic similarity index in Flapjack (Milne et al. 2010). Mean genetic similarity (or the number of shared SNPs) was 80.5% for barley and 65.8% for potato. Genetic similarity was not assessed for faba bean or sweet potato because genotyping data were unavailable.

Barley and faba bean seeds were surface-sterilized in 70% (v/v) ethanol for 30 s, followed by 5% (v/v) sodium hypochlorite for 15 min, and rinsed with sterile water. Seeds were germinated on 0.5% water agar plates at room temperature for 3 days (barley) or 5 days (faba bean). Potato tubers were pre-germinated in an incubator at 20 °C for 7 days prior to transplanting. Sweet potato cuttings were obtained from mother plants, rooted in moist sand trays for 14 days, and then transplanted into pots. Seedlings or plantlets were transplanted into pots containing 3 kg (potato) or 0.8 kg (other crops) of sieved (4 mm) agricultural soils from England (ADAS: clay loam; 53.16625° N, 1.0037492° W), France (Arvalis: silty clay loam; 47.8837° N, 1.5202° E), and Scotland (Bullionfield, James Hutton Institute: silty loam; 56.46111° N, 3.07139° W), with detailed soil properties described in SI Tab. 2. Five replicates per genotype and soil were grown in a completely randomized design in a greenhouse at the James Hutton Institute (UK) with 16 h daylight (min. 200 µmol m⁻2 s⁻1 PAR), 65% humidity, and day/night temperatures of 25 °C/20 °C for sweet potato or 18 °C/14 °C for the other crops. Pots were watered daily with sterilized deionized water to 80% field capacity. Two weeks after planting, 25 ml kg⁻1 soil of zero-nitrogen fertilizer (faba bean) or low-nitrogen fertilizer (other crops) was applied weekly until harvest (SI Tab. 1). These controlled environment conditions were designed to provide non-stress growth conditions, which were supported by the absence of visible deficiency or stress symptoms in the plants throughout the experiment. Plants were harvested before barley and faba bean reached inflorescence (BBCH 32 and 35, respectively) and before tuber formation in potato and sweet potato (before BBCH 40).

### Root exudate sampling

Root exudates were collected 21 days after planting for potato and 35 days for the other crops using a soil-hydroponic hybrid method that enabled investigation of soil effects on root exudation while maintaining the reproducibility and feasibility of hydroponic exudate collection (Oburger and Jones 2018; Santangeli et al. 2024), allowing the large sample sizes required for this study. Rooted soil blocks were carefully removed from pots and adhering soil was gently rinsed from roots with deionised water (DI). As exudate sampling solution, high-quality water (18 MΩ·cm; Thermo Electron LED GmbH, Germany) containing 5 mg L⁻^1^ of bacteriostatic agent (Micropur® Classic MC 1′000F; Katadyn Group, Switzerland) was used to prevent microbial decomposition of exuded metabolites (Otxandorena-Ieregi et al. 2024). Prior to sampling, intact roots were fine-washed and placed for 5 min in the same solution as a pre-rinse step to remove metabolites from damaged cells and to allow osmotic adjustment. Plants were then transferred to final containers with an adapted volume (based on root size) of sampling solution, maintaining a root dry weight (g) to solution volume (mL) ratio of 2–4. Volumes were 125–275 mL for barley, 250 mL for faba bean and potato, and 125 mL for sweet potato. Containers were wrapped in aluminium foil and returned to the greenhouse for 3 h of exudate collection. Because the time between soil removal and exudate sampling was kept short (within 3 h) (Oburger and Jones 2018), measured exudation was assumed to reflect the physiological status established during growth in soil, rather than substantial acclimation to hydroponic conditions during sampling. Thereafter, plants were removed from the solution, shoots and roots were harvested, separated into different aliquots, and weighed (for details see below). Exudates were filtered (0.2 μm cellulose acetate; Chromafil™ CA-20/25 (S), Macherey–Nagel, Germany), aliquoted into 50 mL tubes (Sarstedt, Germany), and frozen at − 20 °C. Aliquots were subsequently freeze-dried before shipping for further analysis (for details see below). The effects of freeze-drying and reconstitution on dissolved organic carbon (DOC) concentrations were evaluated in preliminary trials, with no significant alterations detected.

### Plant growth parameters and root morphological traits

Before root harvest, nodules on the faba bean root systems were counted, and the mother tuber (planted seed tuber) was removed from the intact potato root system. Root biomass was divided into four representative aliquots. First, the root system was longitudinally cut into three sections (upper, middle, lower), and each section was then subdivided into four equal parts, yielding 12 subsections. These 12 subsections were arranged in a 4 × 3 grid and randomly combined into four aliquots, each containing material representative from all root regions (upper, middle, and lower). Two root aliquots were weighed, flash-frozen in liquid nitrogen, and stored at –80 °C for archival purposes and possible future molecular analyses. For this study, a third root aliquot from each crop was weighed and stored in 30% ethanol at 4 °C until analysis of root morphological traits. Fresh weights of the remaining root aliquot and the shoot were recorded before drying at 60 °C for 72 h to determine root and shoot biomass dry weights. Intact root aliquots stored in ethanol were scanned individually (Epson Expression 12000XL; Seiko Epson Corporation, Japan) using the following settings: professional mode, 400 dpi, 8-bit grayscale, high unsharp mask, and increased brightness (50 for most roots, 20 for the darker roots of faba bean). Root scans were analysed with WinRHIZO™ (Pro Version 2017; Regent Instruments, Canada) using a grayscale threshold of 225 (170 for faba bean). Total root length (m), root surface area (cm2), root volume (cm3), and average root diameter (mm) were extrapolated from the scanned aliquot to the entire root system based on the ratio of total to subsample root dry weight. Calculations of further morphological root traits include the specific root length (SRL): total root length/ root dry weight (m g^−1^) and the root tissue density (RTD): root dry weight/ root volume (g cm^−3^).

### Root exudate analysis

Total dissolved organic carbon (DOC) and total dissolved organic nitrogen (DON) concentrations in exudate samples were measured using a Vario Elementar TOC analyzer (Elementar Analysesysteme GmbH, Germany) after re-suspending freeze-dried exudate aliquots (original volume: 20 mL) in 15 mL of HQ water for barley and 10 mL for other crops. To remove inorganic carbonates, 40 µL of 10% HCl was added to 5 mL of the exudate sample prior to measurement. Nitrate concentrations were determined via a colorimetric assay (absorbance: 540 nm; Miranda et al. 2001) and subtracted from total N to calculate DON.

Spectrophotometric assays were used to quantify soluble carbohydrates, amino acids, and phenolic compounds in root exudates using a plate reader (TECAN Infinity® 200 PRO nano, Tecan, Austria) as previously described in Santangeli et al. (2024) and Schwalm et al. (2024). For soluble carbohydrates, the sulfuric acid method (Albalasmeh et al. 2013) was applied to barley, potato, and sweet potato samples (absorbance: 315 nm), while the anthrone colorimetric assay (Hansen and Møller 1975) was used for faba bean samples (absorbance: 625 nm) to avoid background interference. Amino acids were measured using a spectrofluorometric assay (excitation: 340 nm, emission: 450 nm; Jones et al. 2002) without ammonium correction, as ammonium was below detection limits. Phenolics were quantified using a modified Folin-Ciocalteu method (absorbance: 765 nm; Ainsworth and Gillespie 2007). Crop- and compound-specific pre-concentration of exudate samples ensured alignment with calibration curves (SI Tab. 3). Calibration standards included D-glucose for carbohydrates (0.025–1 mM), alanine for amino acids (0.5–50 µM), chlorogenic acid for phenolics (5.6–282 µM), and potassium nitrate for nitrate (3.9–500 µM). Concentrations of DOC, DON, and targeted compounds were corrected for pre-concentration factors and original sampling volumes before calculating exudation rates per cm2 root surface area per hour. Whole-plant exudation rates were additionally obtained by using the corrected concentrations without normalisation to any root metric. Final concentrations of the target compound classes are expressed as equivalents of the respective calibration standards, therefore representing only an approximation of the absolute concentrations of the entire compound class. The relative contribution of each compound class to DOC exuded was estimated based on compound concentrations and the number of C atoms in the calibration standards (Santangeli et al. 2024). Plant C content was estimated as 46.8% of total dry biomass (Ma et al. 2018) to calculate fractional C loss as the daily exudation flux expressed as a percentage of total plant C (SI Tab. 9).

### Statistical analysis

Statistical analyses were conducted in R (version 4.4.0; R Foundation for Statistical Computing). Treatment effects (genotype and soil type) on each individual variable were tested using two-way ANOVA followed by Tukey’s Honest Significant Difference (HSD) test for pairwise comparisons. To evaluate treatment effects on multivariate trait patterns, we additionally performed a Permutational Multivariate Analysis of Variance (PERMANOVA) on sets of variables (e.g., plant growth variables listed in Tab. 1 and exudate variables in Tab. 2). Due to violations of homogeneity of variances for crop comparisons (*n* = 47–58), Welch’s ANOVA with Games–Howell post-hoc tests were applied (see 3.2). Non-parametric Kruskal–Wallis tests with pairwise Wilcoxon rank-sum tests were performed when the data did not meet the assumptions of normality. Pairwise correlations were analysed using Spearman’s rank correlation coefficient to accommodate non-normal data distributions, with multiple testing corrections implemented via the Benjamini–Hochberg procedure to control the false discovery rate (Benjamini and Hochberg 1995). Data were log- or square-root transformed, if required, to meet normality and homoscedasticity assumptions. Means were considered significantly different when the (adjusted) *p*-value was below 0.05. Figures were created in R and BioRender.com, with final edits in Affinity Designer 2 (version 2.3.0) for publication quality.

## Results

### Biomass and root morphological traits

The effects of soil type and genotype on plant growth varied among crops. Main plant growth parameters, including biomass traits, such as shoot dry weight (SDW), root dry weight (RDW), and the root-to-shoot ratio (R:S), as well as root morphological traits, such as specific root length (SRL), root diameter (RD), and root tissue density (RTD) are summarized in Tab. 1 as soil-specific differences (averaged across genotypes within each soil).

**Table 1:** Biomass and root morphological traits of barley (*Hordeum vulgare*), faba bean (*Vicia faba*), potato (*Solanum tuberosum*), and sweet potato (*Ipomoea batatas* (L.) Lam.) (mean ± standard error across four genotypes per crop) grown in the three soils ADAS (AD), Arvalis (ARV), and Bullionfield (BF). PERMANOVA (Euclidean distance) and two-way ANOVA with Tukey’s HSD post-hoc test were used to assess the effects of soil (S), genotype (G), and their interaction (G × S) on biomass and root morphology, allowing evaluation of both multivariate and individual trait responses

		R:S	SDW	RDW	SRL	RD	RTD	PERMANOVA	
			(g)	(g)	(m g^−1^)	(mm)	(g cm^−3^)	R^2^	*p*-value
Barley	AD	0.41 (0.02) a	0.96 (0.08) b	0.38 (0.02) b	288 (7.30) a	0.16 (0.00) b	0.18 (0.01) a		
	ARV	0.35 (0.02) b	1.51 (0.05) a	0.52 (0.02) a	250 (6.77) b	0.17 (0.00) b	0.19 (0.01) a		
	BF	0.41 (0.03) a	1.43 (0.05) a	0.59 (0.03) a	258 (6.47) b	0.19 (0.00) a	0.14 (0.01) b		
	S	*	***	***	**	***	***	0.28	**
	G	***	ns	*	ns	ns	ns	0.03	ns
	SxG	ns	ns	ns	ns	ns	ns	0.04	ns
Faba bean	AD	0.28 (0.02) b	2.11 (0.17) a	0.59 (0.05) a	51.6 (3.52) a	0.51 (0.02) a	0.11 (0.01) a		
	ARV	0.33 (0.02) ab	2.03 (0.19) a	0.66 (0.07) a	49.0 (3.77) a	0.51 (0.01) a	0.11 (0.01) a		
	BF	0.35 (0.03) a	1.97 (0.18) a	0.74 (0.09) a	51.4 (4.32) a	0.55 (0.02) a	0.09 (0.01) a		
	S	*	ns	ns	ns	ns	ns	0.01	ns
	G	**	***	***	ns	***	ns	0.16	*
	SxG	ns	ns	ns	ns	ns	ns	0.06	ns
Potato	AD	0.19 (0.01) a	3.47 (0.25) b	0.62 (0.05) b	139 (7.72) ab	0.32 (0.02) a	0.18 (0.01) a		
	ARV	0.18 (0.01) ab	3.48 (0.24) b	0.64 (0.05) b	129 (6.55) b	0.27 (0.02) a	0.16 (0.01) a		
	BF	0.15 (0.01) b	5.90 (0.39) a	0.88 (0.04) a	144 (6.25) a	0.30 (0.02) a	0.13 (0.00) b		
	S	*	***	***	*	ns	***	0.04	ns
	G	**	***	***	***	***	ns	0.59	***
	SxG	ns	ns	ns	ns	ns	ns	0.07	ns
Sweet potato	AD	0.29 (0.03) a	0.81 (0.09) a	0.22 (0.02) a	106 (9.40) a	0.30 (0.01) b	0.15 (0.01) a		
	ARV	0.21 (0.03) a	0.90 (0.10) a	0.17 (0.02) ab	82.3 (6.51) a	0.34 (0.01) a	0.14 (0.01) a		
	BF	0.24 (0.03) a	0.53 (0.05) b	0.11 (0.01) b	89.7 (7.86) a	0.38 (0.01) a	0.11 (0.01) b		
	S	ns	**	***	ns	**	**	0.08	ns
	G	*	*	**	**	***	ns	0.16	*
	SxG	ns	ns	ns	ns	ns	ns	013	ns

Significance levels: *p* < 0.05 (*), *p* < 0.01 (**), *p* < 0.001 (***), ns not significant. Traits: R:S root-to-shoot ratio, SDW shoot dry weight, RDW root dry weight, SRL specific root length, RD root diameter, RTD root tissue density

In barley, soil type was the only significant factor, explaining 28% of the variation in biomass production and root morphology (PERMANOVA*,* Tab. 1). In contrast, in faba bean, potato, and sweet potato, genotypic differences were the main source of variation, accounting for 16% in faba bean and sweet potato, and 59% in potato, with neither soil or S × G showing significant effects (PERMANOVA, Tab. 1).

Barley genotypes developed on average 34.7% less shoot and 31.5% less root biomass in clay loam soil ADAS compared with silty clay loam Arvalis and silty loam Bullionfield. However, SRL was 13.4% greater in ADAS, driven by the formation of thinner roots (lower RD), which increased root length per unit biomass. In contrast, roots grown in soil Arvalis were thicker (higher RD) and denser (higher RTD), resulting in the smallest SRL, whereas roots in soil Bullionfield were thicker but also had lower tissue density, leading to an intermediate SRL. 

In faba bean, neither biomass or root morphology differed significantly across soils (PERMANOVA, Tab. 1). In contrast, significant genotypic differences were observed. Shoot dry weight was significantly greater in the genotypes Zoran (2.81 g) and Lynx (2.02 g) compared to Ascot (1.73 g) and ILB1814 (1.63 g), and this pattern was reflected in root dry weight, with Zoran (0.89 g) and Lynx (0.79 g) exceeding Ascot (0.51 g) and ILB1814 (0.47 g) (SI Tab. 4). The vigorous genotype Zoran exhibited, on average, a 15.5% smaller root diameter than the other genotypes.

In potato, separate analysis of growth parameters by two-way ANOVA revealed a significant soil effect on biomass production (Tab. 1). Potato plants grown in soil Bullionfield produced 69.8% more shoot biomass and 39.7% more root biomass compared to potatoes grown in soils ADAS and Arvalis. Similar to barley, root tissue density was lower in soil Bullionfield (Tab. 1). Biomass also varied significantly among the four genotypes, with shoot and root dry weights ranging from 2.94 g and 0.58 g in the low-vigour genotype Innovator to 5.75 g and 0.89 g in the vigorous genotype Duke of York. Duke of York also exhibited the largest root diameter (on average 0.35 mm, SI Tab. 4).

Sweet potato, in contrast to potato and barley, developed less biomass in Bullionfield than in the other two soils. Root tissue density was lowest in Bullionfield, consistent with the results for potato and barley. Similar to faba bean and potato, genotypic differences alone accounted for significant variation in all growth parameters (PERMANOVA, Tab. 1). Genotypes Minamiyutaka (0.88 g) and Boniato (0.89 g) outperformed Blesbok (0.64 g) and Ives White (0.58 g) in shoot biomass (SI Tab. 4). This pattern was also observed for root biomass, with Minamiyutaka (0.19 g) and Boniato (0.18 g) exceeding Blesbok (0.11 g) and Ives White (0.17 g). However, Ives White exhibited significantly thicker roots (RD) than the other genotypes (SI Tab. 4).

### Crop-specific exudation patterns

**Fig. 1:**
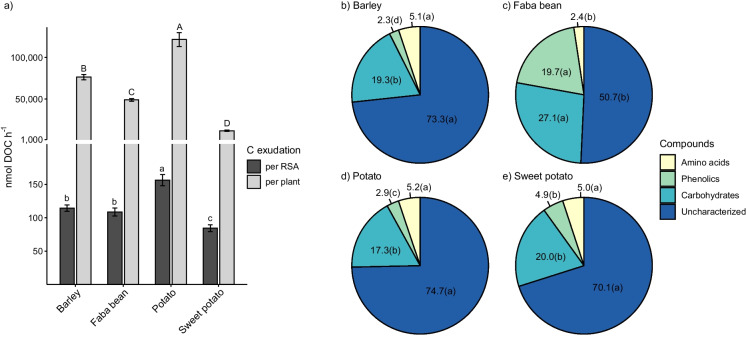
**(a)** Crop-specific dissolved organic carbon (DOC) exudation rates, shown per root surface area (RSA; dark grey) and per plant (light grey). Values are means ± SE, averaged across all genotypes and soil types (*n* = 47–58). Different letters indicate significant differences between crops within each normalisation method (Welch’s ANOVA followed by Games–Howell post-hoc test, *p* < 0.05). (**b-e)** Estimated relative contributions (%) of analysed exudate compound classes to the total DOC exuded by each crop species. Values are mean proportions averaged across all genotypes and soil types. Different letters indicate significant differences between crops within each compound class (Welch’s ANOVA with Games–Howell post-hoc test, *p* < 0.05, *n *= 47–58; see SI Tab. 5)

Total root system DOC exudation differed significantly among crop species, independent of genotype or soil type (Fig. 1a). Potato showed the highest exudation rate per plant (121.5 µmol C plant⁻1 h⁻1), followed by barley (76.2), faba bean (48.8), and sweet potato (11.7). When normalized to root surface area (RSA), C exudation remained significantly highest in potato (152 nmol C cm⁻2 RSA h⁻1), with barley (114) and faba bean (108) releasing comparable amounts, both significantly higher than sweet potato (80.5). Notably, while root surface area–normalized rates were within the same order of magnitude across the crops, whole-plant exudation varied by up to tenfold (potato vs. sweet potato) (Fig. 1a).

Exudation patterns, expressed as the relative contribution of different exudate compound classes to the total DOC exudation rate, differed significantly between the legume faba bean and the three non-legume crops (Fig. 1b-e; SI Tab. 5). Across all crop species, carbohydrates represented the largest identified compound class, accounting for an average of 18.9 ± 0.81% (mean ± SD across potato, sweet potato, and barley), and reaching up to 27% in faba bean. The contribution of carbohydrates to total DOC exudation was 1.4 times greater in faba bean than in non-legumes. Amino acids formed the second-largest targeted compound class in the non-legume crops, contributing an average of 5.1 ± 0.1% to total DOC exudation, but accounted for only 2.4% in faba bean, representing the smallest characterized compound fraction in this species. In contrast to the non-legumes, faba bean exuded a significantly larger proportion of DOC as phenolic compounds, on average 19.7%. This was 8.6, 6.8, and 4 times greater than the phenolic proportion in barley, potato, and sweet potato, respectively. The proportion of DOC as phenolic compounds also significantly differed between the non-legumes, with sweet potato exhibiting 2.1 and 1.7 times greater phenolic exudation than barley and potato, respectively, and potato 1.3 times greater than barley (Fig. 1b-e). Notably, the proportion of uncharacterized exuded DOC was significantly less in faba bean (51%) than in the other crops (70–75%).

### Soil and genotype-driven exudation patterns

A PERMANOVA on total DOC, total DON, carbohydrates, amino acids, phenolics, and uncharacterized compounds was performed to test whether overall exudation profiles differed among genotypes and soil types (Tab. 2; SI Tab. 8). Both, soil type and genotype affected root exudation in faba bean and sweet potato, while exudation in barley was only significantly influenced by soil type, whereas in potato, only genotype played a significant role. Except for barley, genotype was the primary factor driving the variation in exudation rates, explaining 46% of the variation in exudation in faba bean, 27% in sweet potato, 22% in potato. In contrast, soil type contributed to 24% of the variation in barley, to 9% in sweet potato, 7% in faba bean, and showed no significant effect in potato. Interaction effects were only significant for sweet potato, explaining 22% (R^2^ = 0.22) of the exudation variation.

**Table 2:** Overall effect of genotype (G) and soil (S) on exudate parameters including exudation rates per unit root surface area and hour (nmol cm^−2^ h^−1^) of total dissolved organic carbon (DOC), total dissolved organic nitrogen (DON), carbohydrates, amino acids, phenolics, and uncharacterized compounds

	factor	R^2^	*p*-value
Barley	**S**	**0.24**	**0.009**
	G	0.10	0.159
	SxG	0.04	0.909
Faba bean	**S**	**0.07**	**0.048**
	**G**	**0.46**	**0.001**
	SxG	0.08	0.281
Potato	S	0.04	0.273
	**G**	**0.22**	**0.005**
	SxG	0.05	0.716
Sweet potato	**S**	**0.09**	**0.028**
	**G**	**0.27**	**0.002**
	**SxG**	**0.22**	**0.016**

Effect on the exudation variables is considered to be significant when the *p*-value is below 0.05 (bold values). PERMANOVA was conducted based on an Euclidean distance matrix

**Fig. 2:**
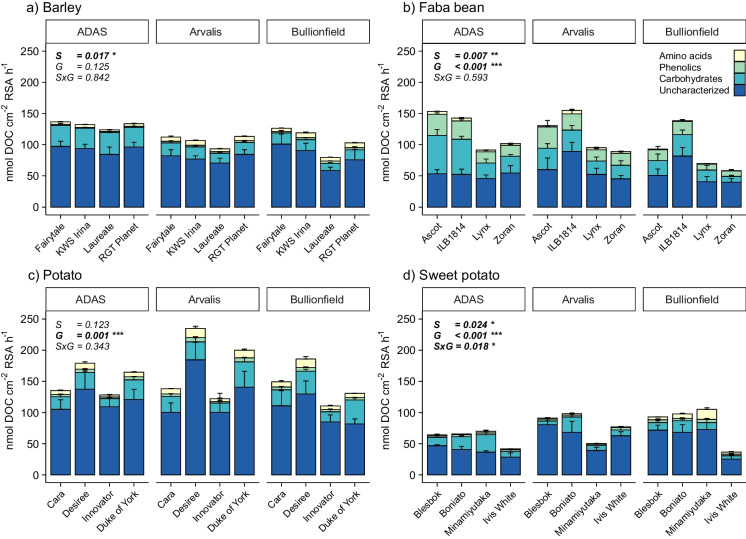
Total dissolved organic carbon (DOC) exudation rates per root surface area per hour, including the estimated contribution of investigated compound classes for (**a**) barley, (**b)** faba bean, (**c)** potato, and (**d**) sweet potato genotypes grown in three different soils (ADAS = clay loam, pH 6.9; Arvalis = silty clay loam, pH 6.9; Bullionfield = silty loam, pH 5.1). Values represent means ± SE, n = 3–5. A two-way ANOVA with Tukey’s HSD post-hoc test was used to investigate the effects of soil (S), genotype (G), and their interaction (SxG) on total DOC exudation rates within each crop. Corresponding p-values are shown in each panel and are reported in SI Tab. 6

Shifting focus from soil- and genotype-driven differences in multivariate exudate profiles to a univariate assessment of the total DOC exudation rates per root surface area (cm2), a two-way ANOVA was applied (Fig. 2). Genotypic differences significantly affected total DOC exudation rates per root surface area (cm^2^) in all crops except barley. In faba bean (Fig. 2b), high-exuding genotypes ILB1814 and Ascot released on average 1.57 times more DOC than Lynx and Zoran. Examination of individual compound classes showed that ILB1814 and Ascot exuded on average 2.02 times more carbohydrates and 1.78 times more phenolics than Lynx and Zoran, whereas amino acid exudation did not differ significantly (SI Tab. 6). In potato (Fig. 2c), total DOC exudation also differed significantly among genotypes. On average, Desiree released significantly more DOC than Cara (1.42-fold) and Innovator (1.66-fold). Patterns for individual compound classes were similar for amino acids, with Desiree releasing 1.70- and 1.91-fold more than Cara and Innovator (SI Tab. 6). Duke of York exhibited the highest carbohydrate exudation rate, exceeding that of Cara by 1.30-fold and Innovator by 2.07-fold. In sweet potato (Fig. 2d), total DOC exudation was significantly affected by genotype, soil, and their interaction (S × G, *p* < 0.05). Therefore, genotype-specific exudation rates were assessed within each soil type (SI Tab. 7). In soil Bullionfield, genotype Minamiyutaka exhibited its highest total DOC exudation rate, largely due to its high amino acid release. In this soil, its amino acid exudation was 3.66-, 2.26-, and 4.67-fold higher than Blesbok, Boniato, and Ives White, respectively (SI Tab. 7). In contrast, Minamiyutaka exhibited the lowest DOC exudation rate among all genotypes in soil Arvalis. In this soil, genotype Ives White increased DOC exudation, reaching levels comparable to the other genotypes, whereas Ives White showed lowest DOC exudation rates in soils ADAS and Bullionfield (SI Tab. 7).

Soil types significantly affected total DOC exudation rates per root surface area (cm^2^) in all crops except potato. In barley (Fig. 2a), total DOC exudation was on average 1.22 times higher in ADAS compared the other soils. Exudation of C assigned to carbohydrates followed this pattern, being 1.79- and 2.06-fold higher in soil ADAS relative to Arvalis and Bullionfield, respectively (SI Tab. 6). In contrast, exudation rates of amino acids and phenolic compounds were lower in ADAS relative to Arvalis (by 67.0% for amino acids, by 69.1% for phenolics), and to Bullionfield (by 74.3% for amino acids, by 99.0% for phenolics). Total DON exudation was generally highest in the slightly acidic soil Bullionfield (SI Fig. 2). In faba bean (Fig. 2b), soil explained only 7% of the variation in exudation (Tab. 2), yet like observed for barley, total DOC exudation rates were still 1.29-fold higher in soil ADAS than in Bullionfield when averaged across genotypes. This pattern was consistent for both carbohydrates and amino acids, which also exhibited higher exudation rates in ADAS than in Bullionfield (SI Tab. 6). In potato (Fig. 2c), exudation rates did not differ across soil types. In sweet potato (Fig. 2d), soil Bullionfield supported higher total DOC, higher DON (SI Fig. 2), and amino acid exudation rates across genotypes compared to ADAS and Arvalis (SI Tab. 6), although these differences depended on genotype (SxG interaction).

### Ordination plots and correlation analysis between biomass, root morphology, and root exudation

**Fig. 3:**
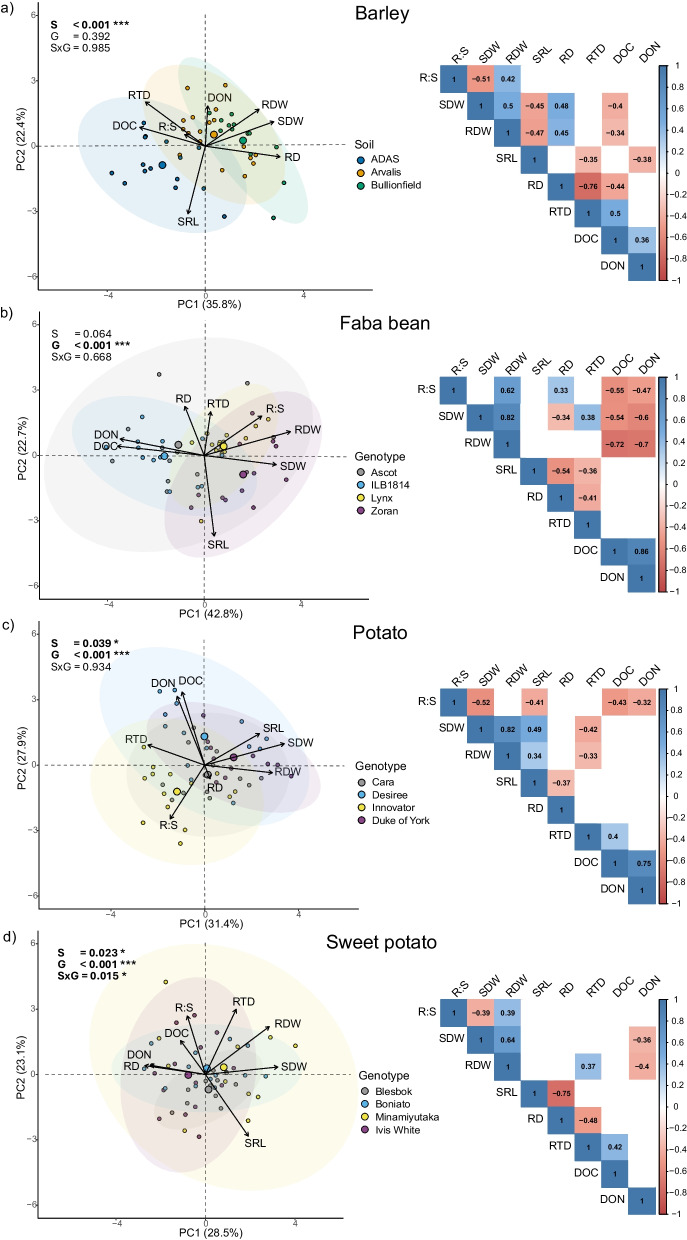
Principal component analysis (PCA) with correlation matrix on biomass, root morphology, and exudation data for (**a**) barley, (**b)** faba bean, **(c)** potato, and (**d**) sweet potato. Correlation matrices show Spearman rank correlations. Non-significant correlations (Benjamini–Hochberg adjusted *p* > 0.05) are omitted. Effects of soil (S), genotype (G), and their interaction (SxG) on the data are indicated by *, **, ***, which refer to 0.05, 0.01, and 0.001 levels of significance, and derived from a PERMANOVA based on Euclidean distance matrix. PCA biplots illustrate the main factor effects for each crop, while minor factor effects are shown in SI Fig. 3. The datasets analysed included root-to-shoot ratio (R:S), shoot dry weight (SDW), root dry weight (RDW), specific root length (SRL), root diameter (RD), root tissue density (RTD), total dissolved organic carbon (DOC) and total dissolved organic nitrogen (DON) exudation rates per root surface area per hour

A selected set of variables was compiled to capture key interactions between exudation, plant biomass, and root morphology. This set included core morphological traits of the Root Economic Space (RES) framework, such as specific root length (SRL), root diameter (RD), and root tissue density (RTD), and was used to visualize the relationships between i) exudation and biomass production and ii) exudation and root morphology using principal component analysis (PCA). A Spearman correlation matrix further confirmed significant correlations (Fig. 3). PCA biplots illustrate the main factor effect for each crop (i.e., soil for barley and genotype for the other crops), while minor factor effects on data distribution are shown in SI Fig. 3.

Plant biomass, measured as shoot and root dry weight, was negatively correlated with exudation rates in all crops except for potato (Fig. 3). Specifically, shoot and root biomass were negatively associated with total DOC exudation in barley, DON exudation in sweet potato, and both DOC and DON exudation in faba bean (Fig. 3a, b, d). In the respective PCA plots, shoot and root biomass were separated from DOC and/or DON exudation rates along the primary axis (PC1), representing a gradient from larger plants with lower exudation rates (per unit root surface area) to smaller plants with higher exudation rates. A Spearman correlation matrix supported these negative relationships in barley, faba bean, and sweet potato (Fig. 3a, b, d). Moreover, significantly negative correlations between R:S ratio and exudation rates of DOC and DON were observed in faba bean and potato (Fig. 3b, c). Exudation rates of assessed compound classes (carbohydrates, amino acids, phenolics) followed the trends of DOC and DON exudation, showing generally negative correlations with biomass and R:S ratio in the respective crops. However, these patterns remained compound-specific across species. For instance, carbohydrate exudation was negatively correlated with barley growth, amino acid exudation with sweet potato growth, and both carbohydrates and amino acids with faba bean growth (SI Fig. 4).

**Fig. 4:**
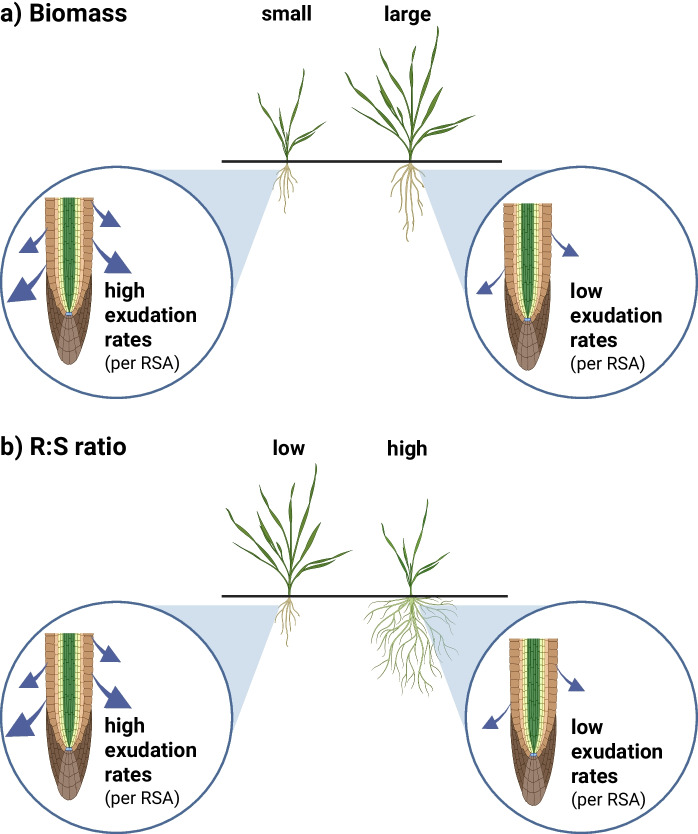
Schematic illustrations are showing the negative correlation between (**a**) biomass and exudation, and (**b**) root-to-shoot ratio (R:S) and exudation. The relationship in (**a**) was confirmed for barley, faba bean, and sweet potato, while (**b**) was confirmed for faba bean and potato. Created in BioRender. Schwalm et al. (2026) https://BioRender.com/xzi3lje

Focusing on the correlation between root morphological traits and exudation rates, we found that root tissue density (RTD) positively correlated with DOC exudation rates in barley, potato, and sweet potato (Fig. 3a, c, d). Only in barley, DOC exudation rate increased with decreasing root diameter (RD), and DON exudation increased with decreasing specific root length (SRL). In faba bean, no significant correlations were observed between root exudation and the core RES traits.

## Discussion

### Crop-specific differences in root exudation

This study explored root exudation under non-stress conditions across crop species, genotypes, and soil types, with crop-specific effects examined first, followed by genotype and soil-type effects. We observed clear differences in total DOC exudation among the four tested crop species (Fig. 1a). As expected, per-plant exudation rates were highest in the species with the largest biomass and root systems. Potato, the largest crop with the most extensive root system, exuded on average 10 times more C per plant than the smallest crop sweet potato, despite having only 4.4 times greater root biomass. This disproportionate response suggests that factors beyond root size alone influence exudation. Normalising DOC exudation per RSA to account for size-independent factors showed a similar pattern to per-plant exudation: potato exuded the most C per cm^2^ RSA, followed by barley and faba bean, all of which released more C than low-exuding sweet potato (Fig. 1a). Exudation rates per unit root were found to decrease with increasing plant developmental stage, especially in annual crops (Aulakh et al. 2001; Santangeli et al. 2024; Schwalm et al. 2024). Considering that potato was grown for only 3 weeks, whereas the other three crops were grown for 5 weeks, this age difference may have contributed to the higher DOC exudation rate per RSA observed in potato. The remaining variation in exudation indicates that, beyond plant age, intrinsic mechanisms such as resource partitioning and trade-offs are likely crop-specific drivers of exudation patterns. Correlations with growth traits (e.g., shoot biomass and R:S ratio) and root morphological traits support this view, suggesting that exudation capacity is governed by C allocation between shoots, roots, and exudates, as discussed in subsequent sections.

Distinct exudation patterns among the four crops were also evident in the composition of compound classes (Fig. 1b–e). The non-legume crops barley, potato, and sweet potato exhibited similar profiles, whereas the legume faba bean showed a significantly different compound composition. In the non-legumes, carbohydrates dominated the DOC fraction, while phenolics and amino acids contributed only minor proportions. In contrast, faba bean released a larger proportion of carbohydrates and also showed a 6.5-fold greater contribution of phenolics to total DOC compared to the non-legumes. This large phenolics proportion may reflect functional roles associated with nitrogen-fixing symbioses. Flavonoids, a phenolic subgroup, have been reported to act as chemo-attractants for rhizobia (Reddy et al. 2007) and as signalling molecules during nodulation (Cooper 2004; Phillips and Tsai 1992) when exuded. Contributions of specific phenolic constituents, such as flavonoids, cannot be resolved with the applied spectrophotometric measurements, and interpretations at this level should therefore be made with caution. Nevertheless, exudation of phenolics measured in our study is within the same order of magnitude as the specific flavonoid exudation reported for faba bean by Liu et al. (2017), who found positive correlations between flavonoid exudation and nodulation. This suggests that the substantially greater phenolic proportion in faba bean exudates relative to the non-legumes may be related to symbiotic signalling processes.

The described patterns in exudate composition are not unique to the species examined here. Dominance of carbohydrates in the characterized fraction of DOC exudation has also been reported for cereals such as maize (Santangeli et al. 2024) and rice (Aulakh et al. 2001; Schwalm et al. 2024), though with proportions closer to faba bean (> 20%) than to the non-legumes investigated here (≤ 20%). However, 51% of DOC in faba bean and 70–75% in the other species fell within the uncharacterized fraction, consistent with values reported in studies using similar spectrophotometric approaches (Santangeli et al. 2024; Schwalm et al. 2024). This substantial uncharacterized exudate pool highlights the complexity of root exudates and points to the opportunity for non-targeted metabolomic approaches to further resolve this fraction. Plausible contributors to this fraction include organic acids, which represented the dominant chemical group in barley and maize exudates relative to carbohydrates and amino acids (Naveed et al. 2017), and accounted for up to 30% of total exuded C in rice, largely as citrate and malate (Schwalm et al. 2024). Metabolomic analyses could further resolve the molecular diversity of the measured compound classes and test whether the relatively large phenolic DOC fraction in faba bean is associated with an increased exudation of specific flavonoids involved in symbiotic signalling (Cooper 2004; Liu et al. 2017).

### Genotype as main driver for variation in exudation – except for barley

As expected (H1), exudation rates varied among agronomically relevant genotypes and soil types. However, their influence differed among crops. Exudation patterns were primarily driven by genotypic differences among lines of faba bean, potato, and sweet potato, with little or no soil effect, except for barley (Tab. 2; Fig. 2). Exudate studies comparing ancestral and modern cultivars reported apparent differences in exudation between these groups (Iannucci et al. 2017; Schwalm et al. 2024), suggesting that distinct exudation profiles are associated with greater genetic divergence. Indeed, variation in root exudation has been linked to such genetic diversity in several species, including wheat, maize, and *Arabidopsis thaliana* (Iannucci et al. 2017; Lopez-Guerrero et al. 2022; Mönchgesang et al. 2016). In our study, genotypic data were available only for barley and potato. Barley genotypes were relatively similar (mean allelic similarity 80.5%) and showed no significant differences in exudation (Tab. 2; Fig. 2), whereas potato genotypes were less similar (65.8%) and exhibited significant genotypic variation in exudation (Tab. 2; Fig. 2). These results hint that greater genetic divergence may contribute to greater variability in exudation. Although genotypic data for faba bean and sweet potato were not available, the pronounced variation in exudation among sweet potato (R^2^ = 0.27, Tab. 2) and particularly faba bean genotypes (R^2^ = 0.46, Tab. 2) indicates that these lines may also differ genetically. Future studies with comprehensive genotyping data are needed to test whether genetic relatedness consistently predicts the magnitude of exudation variation.

Soil type also had a significant impact on exudation patterns, most prominently in barley and to a smaller extent in faba bean and sweet potato, whereas potato remained unaffected (Tab. 2). The absence of a soil type effect in potato could be linked to its shorter growth period (3 weeks), likely being insufficient to manifest soil-dependent differences under non-stress conditions. Interpreting soil effects on exudation is challenging because soils differ in multiple physicochemical parameters that significantly shape plant physiology, thereby making it difficult to disentangle individual drivers of exudation rates. Accordingly, we focused only on the most contrasting soil characteristics, particularly soil organic C content and soil texture. Moreover, most soil-based exudation sampling approaches still collect root exudates hydroponically for a short period of time after removing the roots from their natural soil environment (Maurer et al. 2021; Santangeli et al. 2024). Such approaches do not capture rhizosphere dynamics directly in situ, but instead reflect soil-conditioned legacy effects on plant physiology persisting during short-term hydroponic exudate sampling. As this study employs the same sampling approach, soil effects on exudation are interpreted within this context.

In sweet potato, a clear soil × genotype interaction emerged, as genotype Minamiyutaka exhibited a significantly higher amino acid exudation rate after growth in the C-rich soil Bullionfield (29.1 g C kg⁻1; SI Tab. 2) compared to the other soils. In barley, the highest total DOC and carbohydrate exudation rates occurred in the C-rich clay-loam soil ADAS (23.7 g C kg⁻1; SI Tab. 2). This aligns with a previous study reporting increased exudation of primary metabolites during hydroponic sampling after growth in C-rich compared with C-poor soil (Maurer et al. 2021), suggesting that prior growth in contrasting soils can leave persistent physiological imprints detectable during short-term hydroponic exudate collection. Although higher soil organic C is believed to stimulate exudation in situ via microbial-driven concentration gradients (Canarini et al. 2019), it remains unclear whether this mechanism conditioned the plant physiology to produce the higher exudation rates detected here. Resolving this would require direct evidence from in situ exudate sampling. Our observations, however, do not fully support this hypothesis: barley responded differently in soils ADAS and Bullionfield, despite both being C-rich, and faba bean and sweet potato exhibited similar exudation rates in the C-poor soil Arvalis (12.8 g C kg⁻1; SI Tab. 2) as in the C-rich soils (SI Fig. 1). Taken together, these results suggest that while soil C may contribute, it likely plays a minor role, and other soil characteristics are more important in driving soil-specific exudation patterns.

Differences in soil texture have also been reported to affect root exudation (Neumann et al. 2014). Clay content varied strongly among the soils, with Bullionfield containing only 1.5% clay compared to ADAS (28%) and Arvalis (33%) (SI Tab. 2). The higher clay content of ADAS soil may partly account for the elevated exudation observed in barley after growth in this soil. This pattern is consistent with findings from a field study reporting greater exudation in a clay-rich soil compared to a sandy substrate (Santangeli et al. 2024), which has been attributed to stronger nutrient adsorption onto clay minerals that stimulates exudation as a nutrient mobilization strategy (Chai and Schachtman 2022; Clarholm et al. 2015; Ndzana et al. 2022). The mechanisms underlying higher exudation in clay-rich substrates likely involve multiple soil–plant feedbacks. One possible mechanism is that stronger ethylene accumulation in finely textured soils may constrain root growth (Pandey et al. 2021), potentially reducing C demand for biomass formation and increasing the relative availability of assimilated C for root exudation. Such soil-driven shifts in resource allocation dynamics may explain the elevated exudation rates of barley after growth in the clay-rich soil ADAS compared with the other soil types. The observed trends across soils were nevertheless rather inconsistent with respect to soil textural properties (SI Tab. 2; Fig. 2), indicating that exudation dynamics are governed by a complex interplay of multiple factors. Notably, the relatively limited physicochemical variation among the selected soils may have constrained the magnitude of soil effects observed and limits the broader generalization of our findings.

### Exudation is negatively correlated with biomass and partly with the root-to-shoot ratio

The broadly positive role of root exudation in plant functioning under abiotic stress (Carminati et al. 2016; Chai and Schachtman 2022; Hoffland et al. 2006; Ober et al. 2021) supports the hypothesis that, even under non-stress conditions, increased root exudation rates would be associated with improved plant growth (H2). However, we observed a consistent negative correlation between biomass production and root exudation rates per unit root surface area in all crops except potato, where the correlation was not significant. Expressing exudation as fractional C loss (daily exudation flux as a percentage of total plant C) quantitatively confirmed this negative relationship (SI Tab. 9, SI Fig. 5). The variation in plant size and exudation was driven by the factors soil and genotype (Fig. 3). Larger, more vigorous plants with greater root biomass exuded less, whereas smaller plants exuded more (Fig. 4a). This gradient likely reflects a trade-off in C or N allocation between rhizodeposition and biomass accumulation in barley (C), faba bean (C), and sweet potato (N). C allocation describes the partitioning of photosynthates into different functional plant pools, and the resulting internal competition for available C resources highlights trade-offs among functional sinks (Hartmann et al. 2020), such as those classically described between growth and defence (Herms and Mattson 1992). Our results support the interpretation of root exudation as a C (or N) sink that directly competes with biomass accumulation under non-stress conditions. Under non-stress conditions, sustained high exudation rates may impose a significant cost to the plant, as the C and N allocated to exudates could otherwise support biomass production. In such cases, the benefits of exudation that are advantageous under stress (e.g., enhanced nutrient or water acquisition) may not offset their metabolic costs, ultimately constraining plant growth and explaining the negative correlation between biomass and exudation under non-stress conditions.

We also observed a significant negative correlation between the root-to-shoot ratio (R:S) and DOC and DON exudation rates in faba bean and potato. A low R:S ratio reflects a disproportionately greater shoot relative to root biomass, which may enhance whole-plant photosynthetic assimilation compared to plants with a high R:S ratio. Greater assimilation of photosynthates, for example, in response to nutrient limitation, increases sugar levels in roots, which has been shown to correlate positively with C exudation (Hermans et al. 2006; Juszczuk et al. 2004). Similarly, at low R:S, the enhanced C supply from a well-developed shoot to a comparatively smaller root system may exceed root metabolic and structural demands, resulting in surplus C being released via exudation (Fig. 4b). In this context, the overall lowest R:S ratio observed in potato likely contributed to its highest DOC exudation rates per RSA among the crops (Fig. 1a). This interpretation aligns with the hypothesis that elevated exudation represents a mechanism for surplus C disposal (Prescott et al. 2020), also suggested in rice grown under nutrient limiting conditions (Schwalm et al. 2024). High exudation, therefore, appears less an adaptive strategy for resource acquisition and more a passive consequence of internal C source-sink imbalances. This is not necessarily in disagreement with previously observed increases in exudation of individual metabolites under resource scarcity (Li et al. 2023; Tantriani et al. 2020; Tawaraya et al. 2018). Active upregulation and exudation of a few metabolites may have little effect on the plant’s overall C allocation, which was often not measured in these previous studies. Nevertheless, this process remains only partially addressed in our experiment, as we did not impose growth-limiting conditions. It further does not explain the increased organic N exudation rate at low R:S ratio in faba bean and potato. As variation in R:S ratio, biomass, and exudation was affected by soil and genotype (Fig. 3), these results indicate that the resource allocation trade-offs underlying exudation are driven by both factors.

The observed negative correlation between plant size and exudation is unlikely to be a sampling artifact. In the barley experiment, exudate sampling volume was scaled to root size to maintain a consistent volume-to-root ratio across treatments, ensuring comparable concentration gradients for exudate diffusion (Otxandorena-Ieregi et al. 2024). The persistence of the negative biomass–exudation correlation under these controlled conditions supports the conclusion that it reflects biological allocation strategies rather than methodological bias. Plant developmental stage is another known driver of variation in exudation (Aulakh et al. 2001; Chaparro et al. 2013; Gransee and Wittenmayer 2000). Although we sampled plants at comparable pre-flowering stages, some individuals were close to entering the reproductive phase. This transitional period can involve substantial physiological changes that may influence exudation.

### Correlation between root traits and exudation – morphology traits as proxies for exudation?

Recent studies expanded the RES framework to integrate morphological core traits with exudation characteristics (Kumar et al. 2024; Sun et al. 2021; von Haden et al. 2024; Wen et al. 2022). Building on this perspective, we focused on core morphological RES traits, such as specific root length (SRL), root diameter (RD), and root tissue density (RTD), to assess their relationships with exudation and to evaluate their potential as proxies for estimating root exudation. However, our results indicate that root morphological traits are generally poor proxies for exudation rates (H3), as correlations between morphology and exudation were largely inconsistent or absent across the four crop species under the experimental conditions tested.

Exudation was largely decoupled from the RES collaboration gradient, as exudation rates did not consistently correlate with SRL and RD. In detail, SRL and RD did not reliably predict exudation rates in any crop except barley, where total DOC exudation increased with decreasing RD (Fig. 3). Total DON exudation in barley, however, was associated with greater SRL rather than RD, implying that the relationships between morphology and exudation also depend on the type of exudate. These observations are consistent with previous studies reporting inconsistent correlations between exudation and the morphological core traits SRL and RD. For instance, in maize and grassland species, root exudation was observed to either increase (Santangeli et al. 2024; Williams et al. 2022) or decrease with RD (von Haden et al. 2024). In tree species, exudation increases with SRL (Meier et al. 2020; Wang et al. 2021) and decreases with RD (Jiang et al. 2022; Yin et al. 2023), or no correlation with either trait has been observed (Sun et al. 2021). We initially assumed that contrasting correlations across studies, particularly within the same species, could be explained by differences in exudate sampling methods. However, even with our uniform sampling approach, relationships between exudation and the two traits SRL and RD remained inconsistent or absent. This may be because the collaboration gradient framework conceptualizes RD as a proxy for mycorrhizal accommodation, viewing it as an “outsourcing” strategy for resource acquisition (Bergmann et al. 2020). Accordingly, the presence of arbuscular mycorrhizal fungi (AMF) in the rhizosphere should be considered a prerequisite for evaluating links between exudation and the collaboration gradient. In our study, AMF colonization was negligible in exploratory measurements of a subset of the root samples (SI Tab. 10), which likely contributed to the absence of clear trait–exudation correlations. Notably, the previous studies have collected exudates under conditions decoupled from intact soil, using either cuvette systems (Phillips et al. 2008) or the hydroponic-hybrid approach (Oburger and Jones 2018). Whether morphology–exudation relationships differ under intact soil conditions therefore remains unresolved.

Nevertheless, we consistently observed a positive correlation between DOC exudation rate and RTD across all crops except faba bean, indicating a relationship between exudation and the RES conservation gradient, here represented by RTD as a proxy for tissue conservation (Bergmann et al. 2020). This pattern contrasts with most previous studies, which reported negative associations between RTD and exudation, with denser root tissue linked to less C release in several grassland species (von Haden et al. 2024; Williams et al. 2022), soybean (*Glycine max* (L.) Merr.) (von Haden et al. 2024), and maize (Santangeli et al. 2024; von Haden et al. 2024). Those studies sampled plants after the onset of reproductive growth, whereas our plants were still in the vegetative phase. Vegetative barley plants, also sampled at 35 days after planting, displayed the same positive RTD–exudation correlation observed in our experiments (Otxandorena-Ieregi, in preparation). Similarly, younger maize plants exhibited positive RTD–exudation correlations before shifting to negative later in development (Santangeli, unpublished). This suggests that RTD–exudation relationships change during plant ontogeny. Denser roots early in growth may support higher metabolic activity, leading to greater DOC exudation, whereas in later stages, dense roots may serve primarily for structural support or resource storage, coinciding with reduced metabolic activity and less DOC release. While this remains speculative, follow-up research on the dynamic changes in root functional traits and C allocation over ontogeny will be necessary.

## Conclusion

As expected, exudation rates varied among agronomically relevant genotypes and soil types (H1). Genetic variation among the tested lines was the primary driver of exudation patterns, with greater genotypic diversity resulting in more distinct exudation. Although these initial findings require validation across a larger set of genotypes, they point to opportunities to identify cultivars with favourable exudation traits. Soil type effects were less pronounced than genotype effects, but our results indicate that the influence of both factors on exudation depends on the degree of contrast among the investigated genotypes and soils. While this conclusion may be unsurprising, disentangling the mechanisms underlying genotype- and soil–driven differences in exudation remains challenging due to feedbacks between soil physicochemical properties and plant physiological regulation that dynamically shape exudation patterns.

Root exudation was expected to positively correlate with plant biomass production (H2); however, our findings revealed a contrasting trend under non-stress conditions, with high exudation rates observed alongside reduced plant growth. This suggests a soil and genotype-driven trade-off between biomass accumulation and rhizodeposition, in which exudation benefits do not outweigh their metabolic costs. C allocation between shoots, roots, and exudates emerged as a regulator of exudation capacity, with plant biomass and R:S ratio having a larger influence than root morphology, which proved unreliable as a predictive proxy for exudation rates (H3). By expanding knowledge of genotype- and soil-specific exudation patterns across previously underexplored crops, this study emphasizes that the costs and benefits of exudation are context-dependent. Overall, by revealing how C allocation, genotype, and soil interactions shape root exudation, this study provides new insights for breeding and crop management to harness rhizosphere functions.

## Supplementary Information

Below is the link to the electronic supplementary material.

ESM 1 (PDF 6.24 MB)

## Data Availability

The datasets generated during the current study are available from the corresponding author on reasonable request. R scripts to reproduce figures and statistical analyses are available on GitHub (https://github.com/HenningSchwalm/Soil-and-genotype-driven-root-exudation-patterns-in-barley-faba-beanpotato-and-sweet-potato).

## Abbreviations

C: Carbon
DOC: Dissolved organic carbon
DON: Dissolved organic nitrogen
N: Nitrogen
RD: Root diameter
RES: Root economic space
RSA: Root surface area
RTD: Root tissue density
SRL: Specific root length
