# Supplementary material for: Soil and genotype-driven resource allocation trade-offs shape root exudation across crop species
Source: Plant Soil. 2026 Jun 18;524(2):1269–89. doi: 10.1007/s11104-026-08721-2 (PMC13437571; doi:10.1007/s11104-026-08721-2)
Supplement: Supplementary file 1 [file 11104_2026_8721_MOESM1_ESM.pdf]

Soil and genotype-driven resource allocation trade-offs shape exudation across crop species – Supplementary information

Henning Schwalm[1], Carmen Escudero-Martinez[2,3], Molly Brown[4], Lawrie Brown[4], David Roberts[4], Susan M Mitchell[4], Ignacio Romero Lozano[5], Natacha Bodenhausen[5], Davide Bulgarelli[2], Kelly Houston[4], Timothy S George[4], Eva Oburger[1]

[1] BOKU University, Institute of Soil Research, Department of Ecosystem Management, Climate and Biodiversity, Konrad-Lorenz-Strasse 24, 3430 Tulln an der Donau, Austria; [2] University of Dundee, Plant Sciences, School of Life Sciences, Dundee, United Kingdom; [3] Plant-Microorganism Interaction Department, Institute of Natural Resources and Agrobiology of Salamanca, Consejo Superior de Investigaciones Científicas (IRNASA-CSIC), Salamanca, Spain; [4] James Hutton Institute, Ecological Sciences, Dundee, United Kingdom; [5] FiBL Switzerland, Department of Soil Sciences, Frick, Switzerland

Corresponding author: eva.oburger@boku.ac.at

SI Tab. 1 Total fertilisation of nutrients for barley, potato, and sweet potato after weekly application of 25 ml kg^{-1} soil. Faba bean received the same nutrient solution, except for nitrogen. The agricultural soils used (ADAS, Arvalis, Bullionfield) were not deficient in macro- or micronutrients; therefore, the additionally low fertilisation rate was sufficient to ensure adequate nutrient availability for plant growth.

Element	Chemical	mg kg^{-1} soil
N	$(NH_4)_2SO_4$	35.00
	$Ca(NO_3)$	
	KNO_3	
K	KH_2PO_4	0.73
	KNO_3	
	KCl_2	
P	KH_2PO_4	3.10
Mg	MgO_4S	7.20
Ca	$CaNO_3$	10.0
Ca	$CaCl_2$	9.67
	$CaSO_4 + 2H_20$	0.75
Fe	EDDHA-FeNa / FeNaEDDHA	0.261
	Fe EDTA	0.558
Mn	$MnCl_2$	0.033
B	H_3BO_3	0.023
Zn	$ZnCl_2$	0.004
Cu	$CuSO_4$	0.010
Mo	Na_2MoO_4	0.010
Co	$CoCl_2$	0.006

SI Tab. 2 Physicochemical properties for the used agricultural soils from England (ADAS: clay loam; 53.16625° N, 1.0037492° W), France (Arvalis: silty clay loam; 47.8837° N, 1.5202° E), and Scotland (Bullionfield, James Hutton Institute: silty loam; 56.46111° N, 3.07139° W). CAL-P and CAL-K represent plant-available phosphorus and potassium measured using the calcium-acetate-lactate (CAL) extraction (ÖNORM L1087).

Soil property	ADAS	Arvalis	Bullionfield
pH	6.9	6.9	5.1
Organic C (g kg^{-1} soil)	23.7	12.8	29.1
CAL-P (mg kg^{-1})	118	92.6	73.1
CAL-K (mg kg^{-1})	82.4	87.6	141
Bulk density (g cm^{-3})	0.9	1.2	1.0
Clay (%)	28	33	1.5
Silt (%)	37	62	58.1
Sand (%)	35	5	40.4

SI Tab. 3 Crop and compound-specific pre-concentration of exudate samples to match the concentration ranges of the respective calibration curves. Exudate samples were pre-concentrated by freeze drying 20- and 40-ml sample aliquots and resuspending them in high-quality (HQ) water to obtain the required concentration factor (e.g., freeze-drying 40 ml of faba bean exudate and resuspending in 2 ml HQ water yields a concentration factor of 20, aligning with the calibration range for the phenolic standard chlorogenic acid). Final compound concentrations were corrected by the corresponding concentration factor.

Exudate compound	Crop	Concentration factor
carbohydrates	Barley	1.3
	Faba bean	2.0
	Potato	2.0
	Sweet Potato	2.0
phenolics	Barley	40
	Faba bean	20
	Potato	40
	Sweet Potato	40
amino acids & nitrate	Barley	0.6
	Faba bean	2.0
	Potato	2.0
	Sweet Potato	2.0

SI Tab. 4 Biomass and root morphological traits of barley (*Hordeum vulgare*), faba bean (*Vicia faba*), potato (*Solanum tuberosum*), and sweet potato (*Ipomoea batatas*) (mean ± standard error across three soil types) grown as four different genotypes per crop. PERMANOVA (Euclidean distance) and two-way ANOVA with Tukey's HSD post-hoc test were used to assess the effects of soil (S), genotype (G), and their interaction (G×S) on biomass and root morphology. Significance levels: $p < 0.05$ (*), $p < 0.01$ (**), $p < 0.001$ (***); ns = not significant. Traits: R:S = root:shoot ratio, SDW = shoot dry weight, RDW = root dry weight, SRL = specific root length, RD = root diameter, RTD = root tissue density. Full genotype names: Irina = KWS Irina, Planet = RGT Planet, ILB = ILB1814, York = Duke of York, White = Ives White, Minami.= Minamiyutaka.

		R:S	SDW	RDW	SRL	RD	RTD	PERMANOVA	
			(g)	(g)	(m g^{-1})	(mm)	(g cm^{-3})	R^2	*p*-value
Barley	Fairytale	0.47 (0.03) a	1.17 (0.09) a	0.54 (0.05) a	268 (7.38) a	0.16 (0.01) a	0.18 (0.01) a		
	Irina	0.41 (0.02) ab	1.29 (0.07) a	0.53 (0.03) ab	255 (7.79) a	0.17 (0.00) a	0.18 (0.01) a		
	Laureate	0.33 (0.02) c	1.38 (0.12) a	0.44 (0.03) b	272 (9.45) a	0.18 (0.01) a	0.16 (0.01) a		
	Planet	0.35 (0.02) ac	1.37 (0.10) a	0.47 (0.03) ab	264 (11.5) a	0.17 (0.01) a	0.17 (0.01) a		
	S	*	***	***	**	***	***	0.28	**
	G	***	ns	*	ns	ns	ns	0.03	ns
	SxG	ns	ns	ns	ns	ns	ns	0.04	ns
Faba bean	Ascot	0.28 (0.02) b	1.73 (0.17) b	0.51 (0.08) b	44.4 (3.86) a	0.53 (0.01) a	0.11 (0.01) a		
	ILB	0.28 (0.02) b	1.63 (0.13) b	0.47 (0.04) b	48.7 (2.94) a	0.53 (0.02) a	0.10 (0.00) a		
	Lynx	0.39 (0.02) a	2.02 (0.11) b	0.79 (0.05) a	48.6 (4.15) a	0.57 (0.01) a	0.09 (0.01) a		
	Zoran	0.32 (0.03) ab	2.81 (0.17) a	0.89 (0.08) a	59.6 (3.73) a	0.46 (0.02) b	0.11 (0.01) a		
	S	*	ns	ns	ns	ns	ns	0.01	ns
	G	**	***	***	ns	***	ns	0.16	*
	SxG	ns	ns	ns	ns	ns	ns	0.06	ns
Potato	Cara	0.19 (0.01) ab	3.95 (0.29) b	0.72 (0.06) ab	149 (6.10) a	0.29 (0.02) ab	0.15 (0.01) a		
	Desiree	0.15 (0.01) b	4.56 (0.45) b	0.67 (0.05) ab	153 (5.76) a	0.24 (0.02) b	0.17 (0.01) a		
	Innovator	0.20 (0.01) a	2.94 (0.32) c	0.58 (0.05) b	100 (4.10) b	0.31 (0.02) ab	0.15 (0.01) a		
	York	0.16 (0.01) b	5.75 (0.43) a	0.89 (0.06) a	150 (5.69) a	0.35 (0.03) a	0.15 (0.01) a		
	S	*	***	***	*	ns	***	0.04	ns
	G	**	***	***	***	**	ns	0.59	***
	SxG	ns	*	ns	ns	ns	ns	0.07	ns
Sweet potato	Blesbok	0.19 (0.03) b	0.64 (0.11) ab	0.11 (0.02) b	109 (7.54) a	0.30 (0.01) b	0.14 (0.01) a		
	Boniato	0.21 (0.01) b	0.89 (0.08) a	0.18 (0.02) a	84.5 (4.42) ab	0.34 (0.01) b	0.13 (0.01) a		
	Minami.	0.24 (0.04) b	0.88 (0.11) a	0.19 (0.03) a	105 (10.6) ab	0.31 (0.02) b	0.14 (0.01) a		
	White	0.30 (0.03) a	0.59 (0.07) b	0.17 (0.02) ab	80.9 (7.10) b	0.39 (0.02) a	0.12 (0.01) a		
	S	ns	**	***	ns	**	**	0.08	ns
	G	*	*	**	**	***	ns	0.16	*
	SxG	ns	ns	ns	ns	ns	ns	0.13	ns

SI Tab. 5 Significant differences in the relative contributions of the exudate compound classes to the total dissolved organic carbon (DOC) exuded among the crop species barley, faba bean, potato, and sweet potato ($p < 0.05$, $n = 47$ -58). Differences in normalized relative values of exudate compounds among crop were assessed using Welch's ANOVA to account for unequal variances. Different letters indicate significant differences between crop species within each compound class based on a Games Howell post hoc test. *For carbohydrates, global post hoc tests did not detect a significant difference between faba bean and sweet Potato (Games Howell, $p > 0.05$), despite a large observed difference in normalized mean values. To further investigate, focused pairwise Welch's t-tests and Wilcoxon tests were performed, revealing a significant difference between these two crops (Welch t-test, t = 2.58, df = 95, $p = 0.0115$; Wilcoxon test, $p = 0.0078$), with an estimated mean difference of 7.1%.

Relative contribution to DOC exuded (%)	*p*-value Welch's ANOVA Difference between crops	Games Howell Crop	Games Howell Significance
carbohydrates	< 0.001 ***	Barley	b
		Faba bean	a
		Potato	b
		Sweet Potato	b*
phenolics	< 0.001 ***	Barley	d
		Faba bean	a
		Potato	c
		Sweet Potato	b
amino acids	< 0.001 ***	Barley	a
		Faba bean	b
		Potato	a
		Sweet Potato	a
uncharacterized	< 0.001 ***	Barley	a
		Faba bean	b
		Potato	a
		Sweet Potato	a

SI Tab. 6 *P*-values in bold, derived from a two-way ANOVA, indicate significant effects of soil type (S), genotype (G), and their interaction (SxG) on total dissolved organic carbon (DOC), total dissolved organic nitrogen (DON) exudation rates, as well as on compound group-derived DOC exudation rates (carbohydrates, amino acids, phenolics, and uncharacterized compounds) per unit root surface area in the four crop species studied ($p < 0.05$). Different letters indicate significant differences among soil types (AD = ADAS, clay loam, pH 6.9; ARV = Arvalis, silty clay loam, pH 6.9; BF = Bullionfield, silty loam, pH 5.1) and among genotypes based on Tukey's HSD post-hoc test. In sweet potato, DOC* and amino acid* exudation were significantly affected by the S×G interaction. Therefore, simple effects (genotype-specific rates within soils and soil-specific rates within genotypes) were further examined using post-hoc tests (SI Tab. 6).

	Exudation rate	*p*-value ANOVA			Tukey's HSD			
	(nmol C cm^{-2} RSA h^{-1})	S	G	SxG	Soil type	Significance	Genotype	Significance
Barley	DOC	**0.017**	0.125	0.842	AD	a	Fairytale	a
					ARV	b	KWS Irina	a
					BF	b	Laureate	a
							RGT Planet	a
	DON	**0.002**	0.134	0.998	AD	b	Fairytale	a
					ARV	b	KWS Irina	a
					BF	a	Laureate	a
							RGT Planet	a
	carbohydrates	**< 0.001**	0.723	0.846	AD	a	Fairytale	a
					ARV	b	KWS Irina	a
					BF	b	Laureate	a
							RGT Planet	a
	amino acids	**< 0.001**	**0.024**	0.684	AD	b	Fairytale	ab
					ARV	a	KWS Irina	a
					BF	a	Laureate	b
							RGT Planet	ab
	phenolics	**< 0.001**	0.284	0.293	AD	b	Fairytale	a
					ARV	a	KWS Irina	a
					BF	a	Laureate	a
							RGT Planet	a
	uncharacterized	0.160	0.107	0.703	AD	a	Fairytale	a
					ARV	a	KWS Irina	a
					BF	a	Laureate	a
							RGT Planet	a
Faba bean	DOC	**0.007**	**< 0.001**	0.593	AD	a	Ascot	a
					ARV	ab	ILB1814	a
					BF	b	Lynx	b
							Zoran	b
	DON	0.089	**< 0.001**	0.657	AD	a	Ascot	a
					ARV	a	ILB1814	a
					BF	a	Lynx	c
							Zoran	
	carbohydrates	**< 0.001**	**< 0.001**	0.328	AD	a	Ascot	ab
					ARV	b	ILB1814	a
					BF	b	Lynx	b
							Zoran	b
	amino acids	**< 0.001**	0.056	0.168	AD	a	Ascot	a
					ARV	ab	ILB1814	a
					BF	b	Lynx	a
							Zoran	a
	phenolics	**< 0.001**	**< 0.001**	0.874	AD	a	Ascot	a
					ARV	ab	ILB1814	a
					BF	b	Lynx	b
							Zoran	b
	uncharacterized	0.387	**0.003**	0.392	AD	a	Ascot	ab
					ARV	a	ILB1814	a
					BF	a	Lynx	b
							Zoran	b
Potato	DOC	0.123	**< 0.001**	0.343	AD	a	Cara	b
					ARV	a	Desiree	a
					BF	a	Innovator	b
							Duke of York	ab
	DON	0.294	**0.030**	0.392	AD	a	Cara	ab
					ARV	a	Desiree	a
					BF	a	Innovator	b
							Duke of York	ab
	carbohydrates	0.111	**< 0.001**	0.938	AD	a	Cara	bc
					ARV	a	Desiree	ab
					BF	a	Innovator	c
							Duke of York	a
	amino acids	0.0953	**< 0.001**	0.709	AD	a	Cara	b
					ARV	a	Desiree	a

					BF	a	Innovator	b
							Duke of York	ab
	phenolics	0.502	**< 0.001**	0.492	AD	a	Cara	ab
					ARV	a	Desiree	a
					BF	a	Innovator	b
							Duke of York	a
	uncharacterized	0.156	**0.020**	0.608	AD	a	Cara	ab
					ARV	a	Desiree	a
					BF	a	Innovator	b
							Duke of York	ab
Sweet Potato	DOC*[1]	**0.024**	**< 0.001**	**0.018**	AD	b	Blesbok	a
					ARV	ab	Boniato	a
					BF	a	Minimiyutaka	a
							Ivis White	b
	DON	**0.001**	0.970	0.683	AD	b	Blesbok	a
					ARV	b	Boniato	a
					BF	a	Minimiyutaka	a
							Ivis White	a
	carbohydrates	0.182	**0.004**	0.236	AD	a	Blesbok	b
					ARV	a	Boniato	a
					BF	a	Minimiyutaka	ab
							Ivis White	b
	amino acids*[2]	**< 0.001**	**< 0.001**	**< 0.001**	AD	b	Blesbok	b
					ARV	b	Boniato	b
					BF	a	Minimiyutaka	a
							Ivis White	b
	phenolics	0.151	0.809	0.161	AD	a	Blesbok	a
					ARV	a	Boniato	a
					BF	a	Minimiyutaka	a
							Ivis White	a
	uncharacterized	**0.012**	**0.015**	0.129	AD	b	Blesbok	a
					ARV	a	Boniato	a
					BF	a	Minimiyutaka	ab
							Ivis White	b

SI Tab. 7 Due to the significant S×G interaction detected for DOC*[1] and amino acids*[2] exudation rates per cm^2 root surface area per hour in sweet potato, additional post-hoc analyses were conducted. Comparisons were made among genotypes within each soil (AD = ADAS, clay loam, pH 6.9; ARV = Arvalis, silty clay loam, pH 6.9; BF = Bullionfield, silty loam, pH 5.1) and among soils within each genotype. Different letters indicate significant differences according to Tukey's HSD post-hoc test. Bold values highlight significant differences.

Genotype-specific exudation rates within each soil

Soil	Genotype	DOC	amino acid
AD	Ivis White	**48.7 ± 11.5 b**	1.33 ± 0.91 a
	Blesbok	**68.8 ± 12.8 a**	1.58 ± 1.02 a
	Boniato	**70.6 ± 11.5 a**	1.18 ± 1.02 a
	Minamiyutaka	**84.1 ± 11.5 a**	2.54 ± 1.02 a
ARV	Ivis White	76.7 ± 11.5 ab	1.21 ± 1.00 a
	Blesbok	86.9 ± 12.8 ab	1.74 ± 1.02 a
	Boniato	**113 ± 11.5 a**	2.05 ± 0.91 a
	Minamiyutaka	**57.9 ± 14.8 b**	1.34 ± 1.17 a
BF	Ivis White	**40.6 ± 11.5 b**	**3.40 ± 0.91 c**
	Blesbok	**103 ± 12.9 a**	**4.34 ± 1.02 bc**
	Boniato	**110 ± 11.5 a**	**7.02 ± 0.91 b**
	Minamiyutaka	**114 ± 12.8 a**	**15.9 ± 1.02 a**

Soil-specific exudation rates for each genotype

Genotype	Soil	DOC	amino acid
Ivis White	AD	48.7 ± 11.5 a	**1.33 ± 0.91 b**
	ARV	76.7 ± 11.5 a	**1.21 ± 1.00 b**
	BF	40.6 ± 11.5 a	**3.40 ± 0.91 a**
Blesbok	AD	68.9 ± 12.8 a	1.58 ± 1.02 a
	ARV	86.9 ± 12.8 a	1.74 ± 1.02 a
	BF	103 ± 12.8 a	4.34 ± 1.02 a
Boniato	AD	**70.6 ± 11.5 b**	**1.18 ± 1.02 b**
	ARV	**113 ± 11.5 a**	**2.05 ± 0.91 b**
	BF	**110 ± 11.5 a**	**7.02 ± 0.91 a**
Minamiyutaka	AD	84.1 ± 11.5 ab	**2.54 ± 1.02 b**
	ARV	**57.9 ± 14.8 b**	**1.34 ± 1.17 b**
	BF	**114 ± 12.8 a**	**15.9 ± 1.02 a**

SI Tab. 8 Absolute exudation rates (mean ± standard error) per cm² root surface area per hour of total dissolved organic carbon (DOC), dissolved organic nitrogen (DON), carbohydrates, amino acids, phenolics, and uncharacterized compounds of each crop species grown in three soil types (AD = ADAS, clay loam, pH 6.9; ARV = Arvalis, silty clay loam, pH 6.9; BF = Bullionfield, silty loam, pH 5.1). Carbohydrates expressed in glucose equivalents, amino acids expressed in alanine equivalents and phenolic compounds expressed in chlorogenic acid equivalents. Values are averaged across genotypes. Exudation rates represent absolute compound amounts (nmol), not C-derived quantities per compound. Significant effects of soil (S), genotype (G), and their interaction (S × G) are indicated by * ($p < 0.05$), ** ($p < 0.01$), and *** ($p < 0.001$), based on two-way ANOVA and PERMANOVA (Euclidean distance). ns = not significant.

		DOC (nmol)	DON (nmol)	carbohydrates (nmol)	amino acids (nmol)	phenolics (nmol)	uncharacterized (nmol)	PERMANOVA R2	PERMANOVA *p*-value
	AD	132 (5.75) a	5.07 (0.26) b	5.56 (0.35) a	0.75 (0.06) b	0.10 (0.01) b	15.5 (0.72) a		
	ARV	106 (5.09) b	5.57 (0.33) b	3.11 (0.18) b	1.25 (0.11) a	0.17 (0.01) a	13.1 (0.63) a		
	BF	109 (8.83) b	7.52 (0.64) a	2.69 (0.24) b	1.30 (0.12) a	0.20 (0.01) a	13.1 (1.11) a		
Barley									
	S	*	**	***	***	***	ns	0.24	**
	G	ns	ns	ns	*	ns	ns	0.10	ns
	SxG	ns	ns	ns	ns	ns	ns	0.04	ns
	AD	122 (8.32) a	6.28 (0.66) a	6.92 (0.87) a	0.72 (0.07) a	1.52 (0.13) a	8.66 (0.57) a		
	ARV	108 (10.3) ab	5.84 (0.67) a	4.40 (0.66) b	0.65 (0.13) ab	1.41 (0.14) ab	9.34 (1.20) a		
	BF	94.5 (11.4) b	5.01 (0.79) a	3.92 (0.74) b	0.18 (0.03) b	0.98 (0.12) b	9.07 (1.21) a		
Faba bean									
	S	**	ns	***	***	***	ns	0.07	*
	G	***	***	***	ns	***	**	0.46	***
	SxG	ns	ns	ns	ns	ns	ns	0.08	ns
	AD	152 (12.3) a	8.30 (0.75) a	3.84 (0.40) a	1.32 (0.17) a	0.25 (0.02) a	19.7 (1.65) a		
	ARV	174 (18.9) a	10.5 (1.17) a	4.49 (0.55) a	1.97 (0.28) a	0.30 (0.04) a	22.1 (2.47) a		
	BF	144 (12.1) a	9.02 (0.66) a	4.88 (0.43) a	1.70 (0.26) a	0.28 (0.03) a	16.9 (1.53) a		
Potato									
	S	ns	ns	ns	ns	ns	ns	0.04	ns
	G	***	*	***	***	***	*	0.22	**
	SxG	ns	ns	ns	ns	ns	ns	0.05	ns
	AD	70.3 (6.14) b	4.50 (1.00) b	2.96 (0.54) a	0.35 (0.07) b	0.19 (0.02) a	6.23 (0.50) b		
	ARV	91.3 (7.72) ab	6.44 (1.31) b	2.29 (0.56) a	0.34 (0.09) b	0.18 (0.02) a	10.6 (1.19) a		
	BF	90.0 (10.4) a	14.2 (2.30) a	2.07 (0.33) a	1.48 (0.28) a	0.25 (0.04) a	9.43 (1.27) a		
Sweet potato									
	S	*	***	ns	***	ns	*	0.09	*
	G	***	ns	**	***	ns	*	0.27	**
	SxG	*	ns	ns	***	ns	ns	0.22	*

SI Tab. 9 Fractional C loss (% plant C exuded day⁻¹) per crop, calculated as (mg C exuded plant^{-1} h^{-1}) / (mg C plant^{-1}) × 100 × 24. Plant C content (mg C plant^{-1}) was estimated from total biomass (shoot + root dry weight) assuming a fixed C fraction of 46.8% of dry mass, consistent with published mean values across plant organs (Ma et al. 2018). Mean fractional C losses range from 0.38% d^{-1} in sweet potato to 2.68% d^{-1} in barley, falling within the biologically plausible range of <5% d^{-1} reported in the literature (Badri and Vivanco 2009; Jones et al. 2009; Dilkes et al. 2004). Correlation between fractional C loss and biomass is shown in SI Fig. 5.

Crop	Mean (% C day^{-1})	Min (% C day^{-1})	Max (% C day^{-1})
Barley	2.68	1.01	4.35
Faba bean	0.57	0.23	1.09
Potato	0.65	0.19	1.46
Sweet potato	0.38	0.14	1.01

SI Tab. 10 Arbuscular mycorrhizal fungi (AMF) expressed in % of root length containing AMF structures (hyphae, arbuscules, and/ or vesicles) for one plant per crop grown in three agricultural soils: ADAS (clay loam, pH 6.9), Arvalis (silty clay loam, pH 6.9), and Bullionfield (silty loam, pH 5.1). Data is exploratory and based on a single replicate. Roots were cleared in 1% KOH, acidified in 5% acetic acid, stained with ink–vinegar, destained, and stored in glycerine prior to microscopic assessment.

Soil	Barley	Faba bean	Potato	Sweet Potato
ADAS	0%	0.25%	0%	2.75%
Arvalis	1%	8.50%	1%	7.25%
Bullionfield	1.50%	1.25%	1.50%	7.25%
Average	0.83%	3.33%	0.83%	5.75%

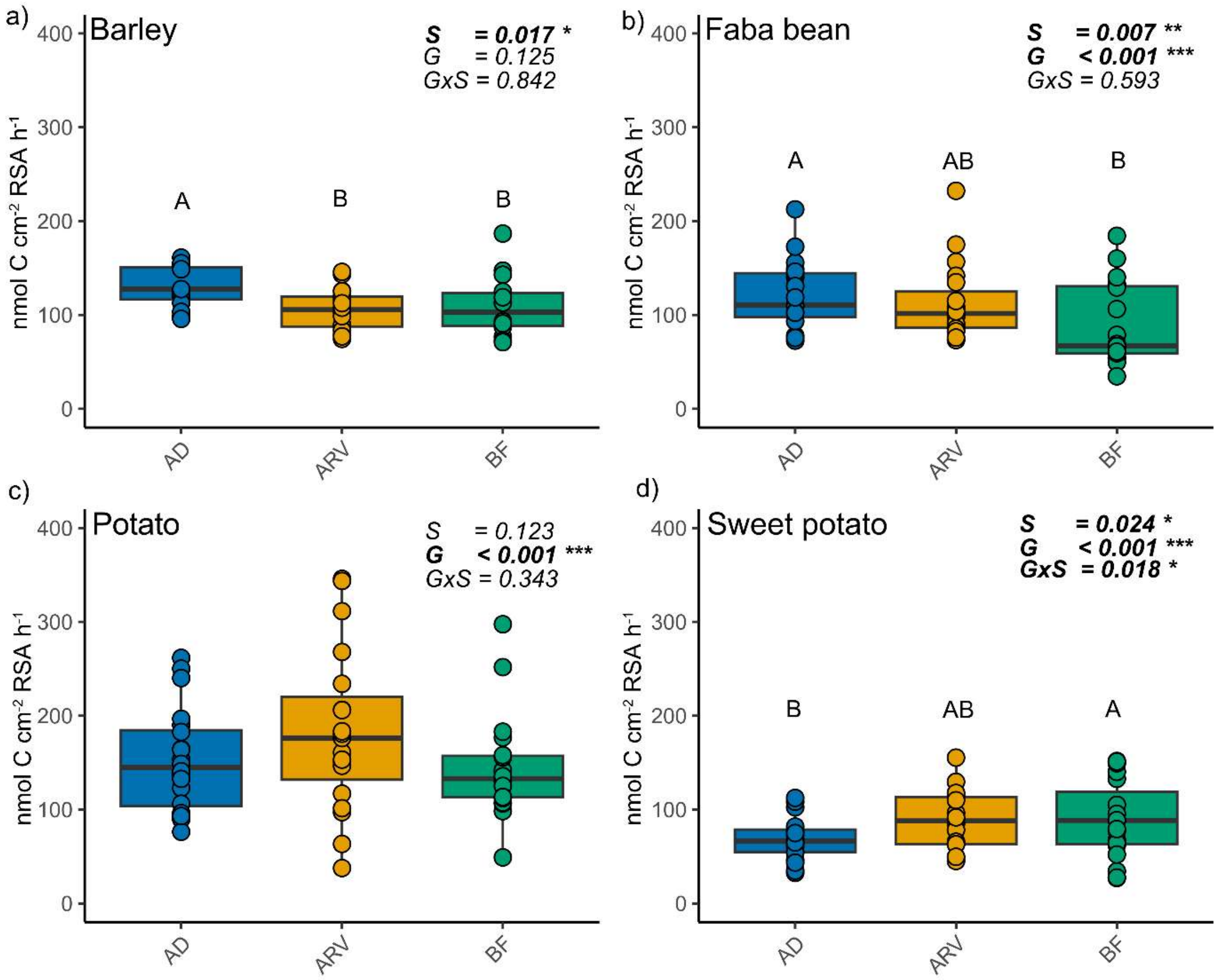

SI Fig. 1 Soil effect on total C exudation rates per root surface area per hour in a) barley, b) faba bean, c) potato, and d) sweet potato. Soil types: AD = ADAS, clay loam, pH 6.9; ARV = Arvalis, silty clay loam, pH 6.9; BF = Bullionfield, silty loam, pH 5.1. Values represent means ± SE, *n* = 5. A two-way ANOVA was employed to investigate the effects of soil (S), genotype (G), and their interaction (GxS) on total C exudation rates.

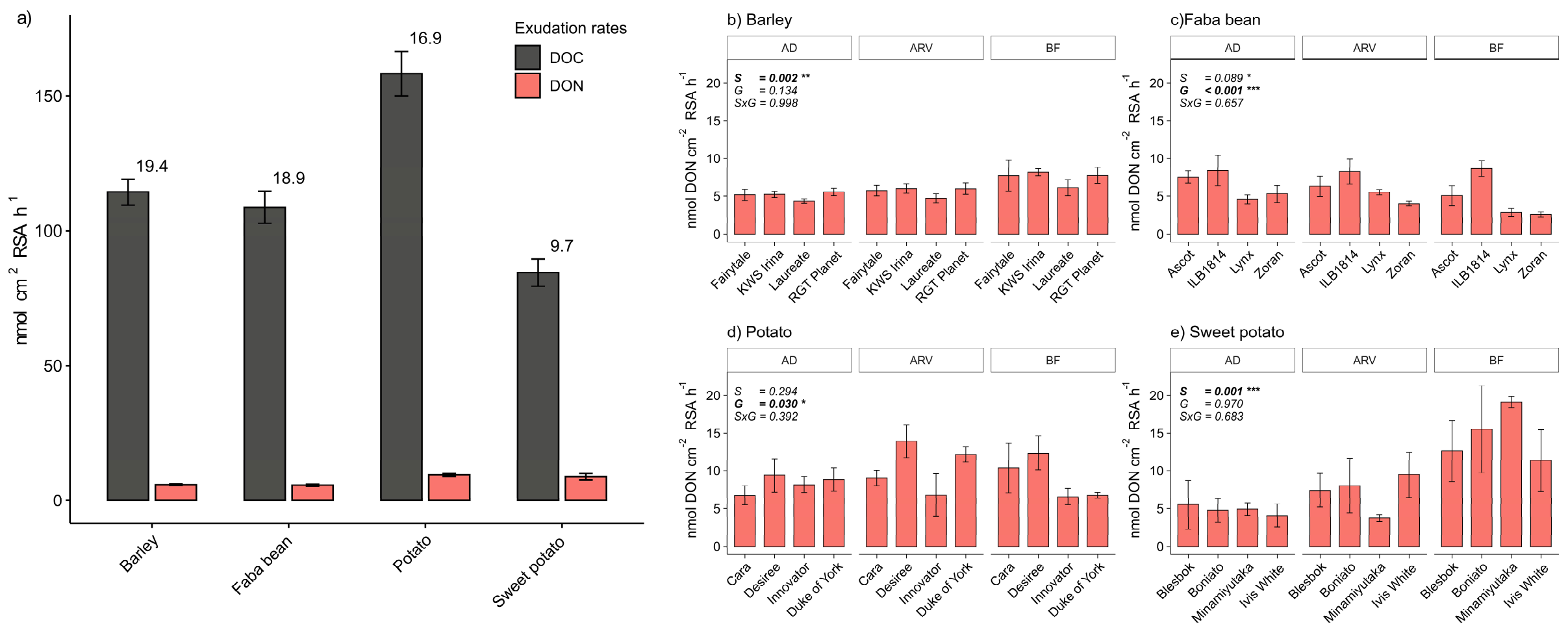

SI Fig. 2 Values above bar charts show the C:N ratio of the exudation per crop. Organic N exudation rates per root surface area for **b)** barley, **c)** faba bean, **d)** potato, and **e)** sweet potato genotypes grown in three different soils (AD = ADAS, clay loam, pH 6.9; ARV = Arvalis, silty clay loam, pH 6.9; BF = Bullionfield, silty loam, pH 5.1). Values represent means ± SE, n = 3-5. A two-way ANOVA with Tukey's HSD post-hoc test was employed to investigate the effects of soil (S), genotype (G), and their interaction (SxG) on organic N exudation rates.

91

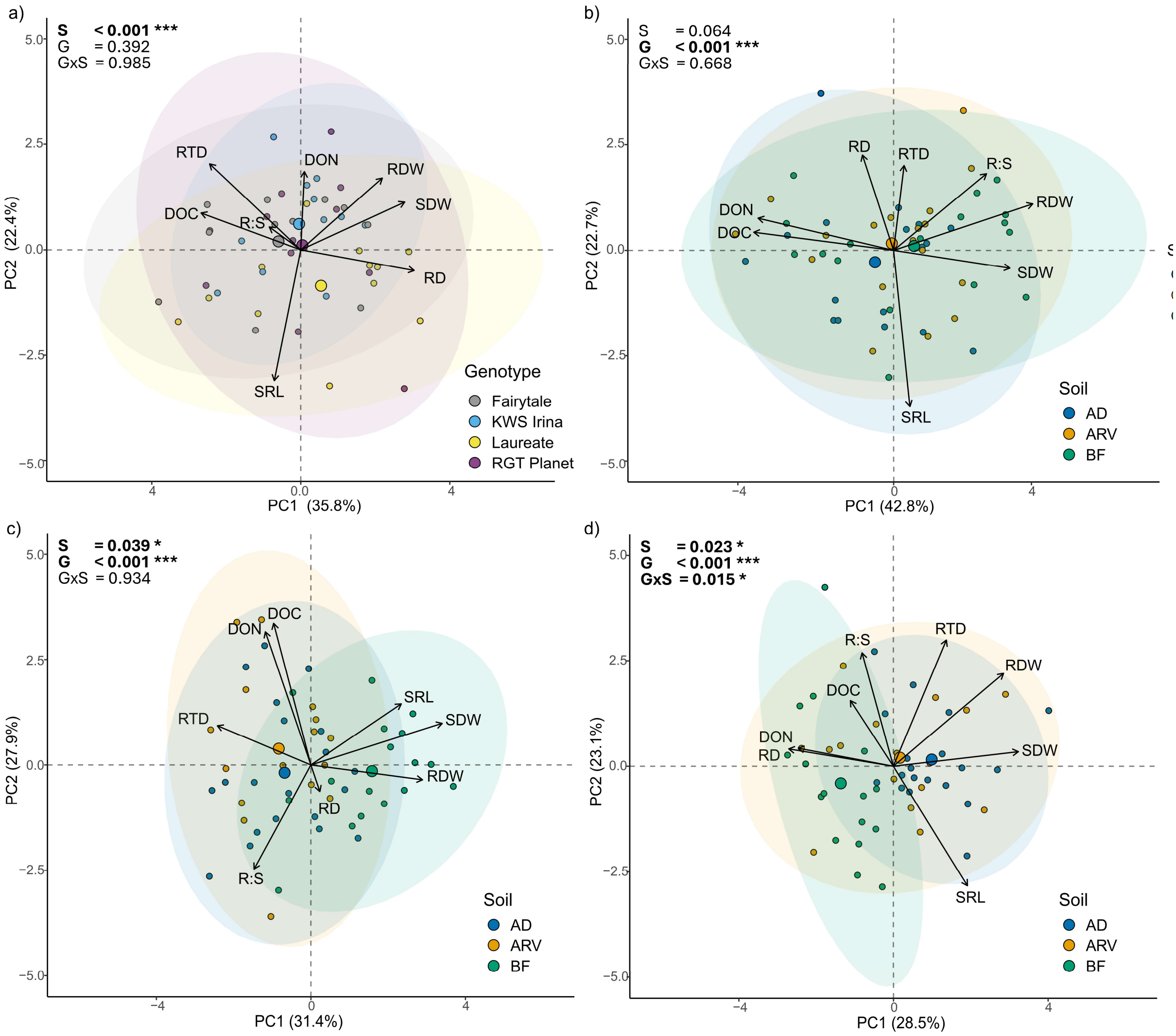

SI Fig. 3 Principal component analysis (PCA) on biomass, root morphology, and exudation data of **a)** barley, **b)** faba bean, **c)** potato, and **d)** sweet potato. Soil (S), genotype (G), and their interaction (SxG) effects on the data are indicated by *, **, ***, which refer to 0.05, 0.01, and 0.001 levels of significance, derived from a PERMANOVA based on Euclidean distance matrix. PCA biplots illustrate minor factor effects for each crop, while the main factor effects are presented in Fig. 3. The dataset analysed included root: shoot ratio (R:S), shoot dry weight (SDW), root dry weight (RDW), specific root length (SRL), root diameter (RD), root tissue density (RTD), total carbon (C ex) and organic N (N_{org}) exudation rates per root surface area per hour. AD = ADAS, ARV = Arvalis, BF = Bullionfield.

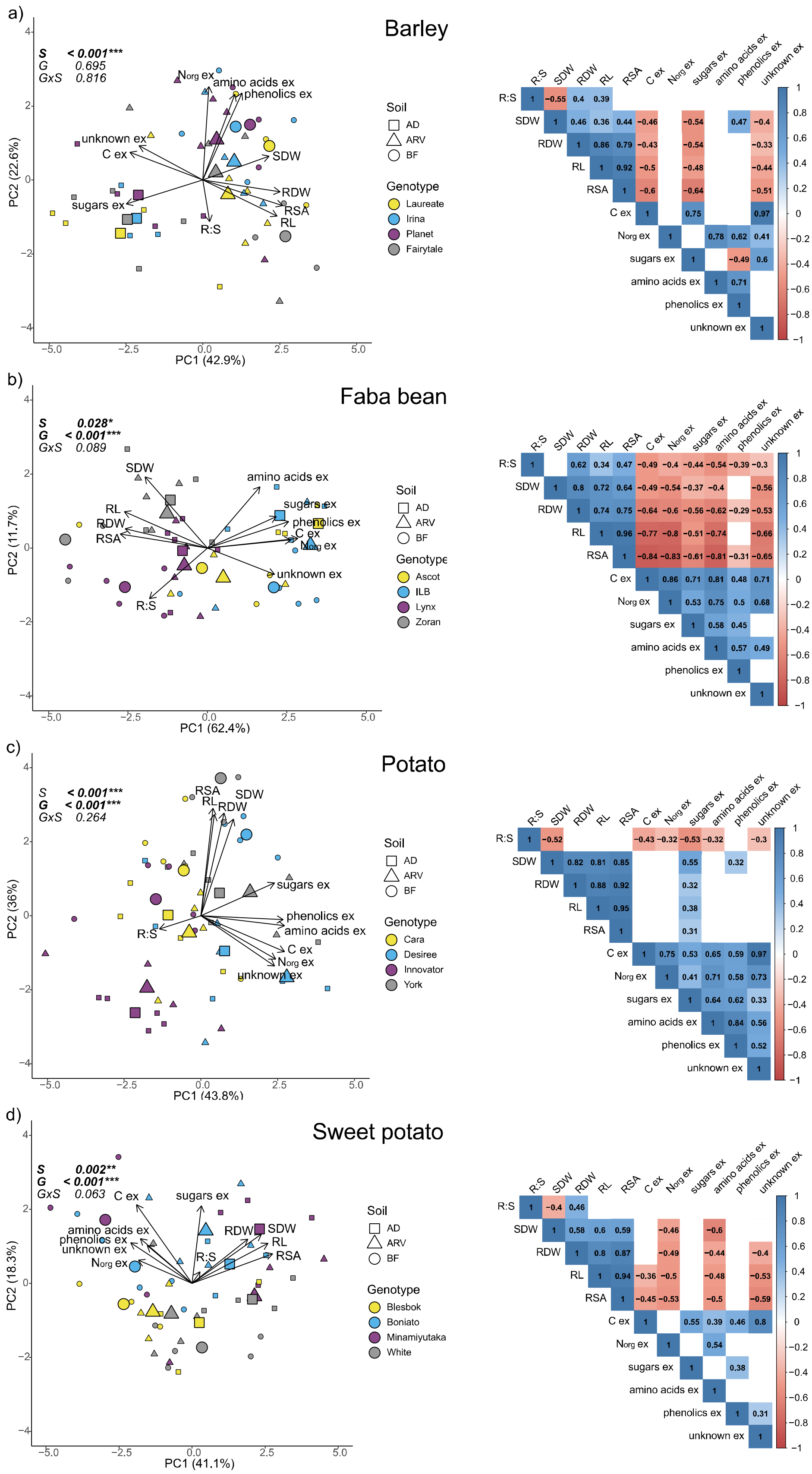

SI Fig. 4 Principal component analysis (PCA) with correlation matrix on plant growth and exudation data for **a)** barley, **b)** faba bean, **c)** potato, and **d)** sweet potato. Correlation matrix showing Spearman rank correlations. Non-significant correlations (Benjamini–Hochberg adjusted p > 0.05) are omitted. Soil (S), genotype (G), and their interaction (SxG) effects on the data are indicated by *, **, ***, which refer to 0.05, 0.01, and 0.001 levels of significance which derived from a PERMANOVA based on Bray-Curtis distance matrix. The dataset analysed included root:shoot ratio (R:S), shoot dry weight (SDW), root dry weight (RDW), total root length (RL), root surface area (RSA), total carbon (C ex), organic N (N_{org}), sugars, amino acids, phenolics, and unknown compounds exudation rates per root surface area per hour. AD = ADAS, ARV = Arvalis, BF = Bullionfield. Full genotype names: Irina = KWS Irina, Planet = RGT Planet, ILB = ILB1814, York = Duke of York, White = Ives White.

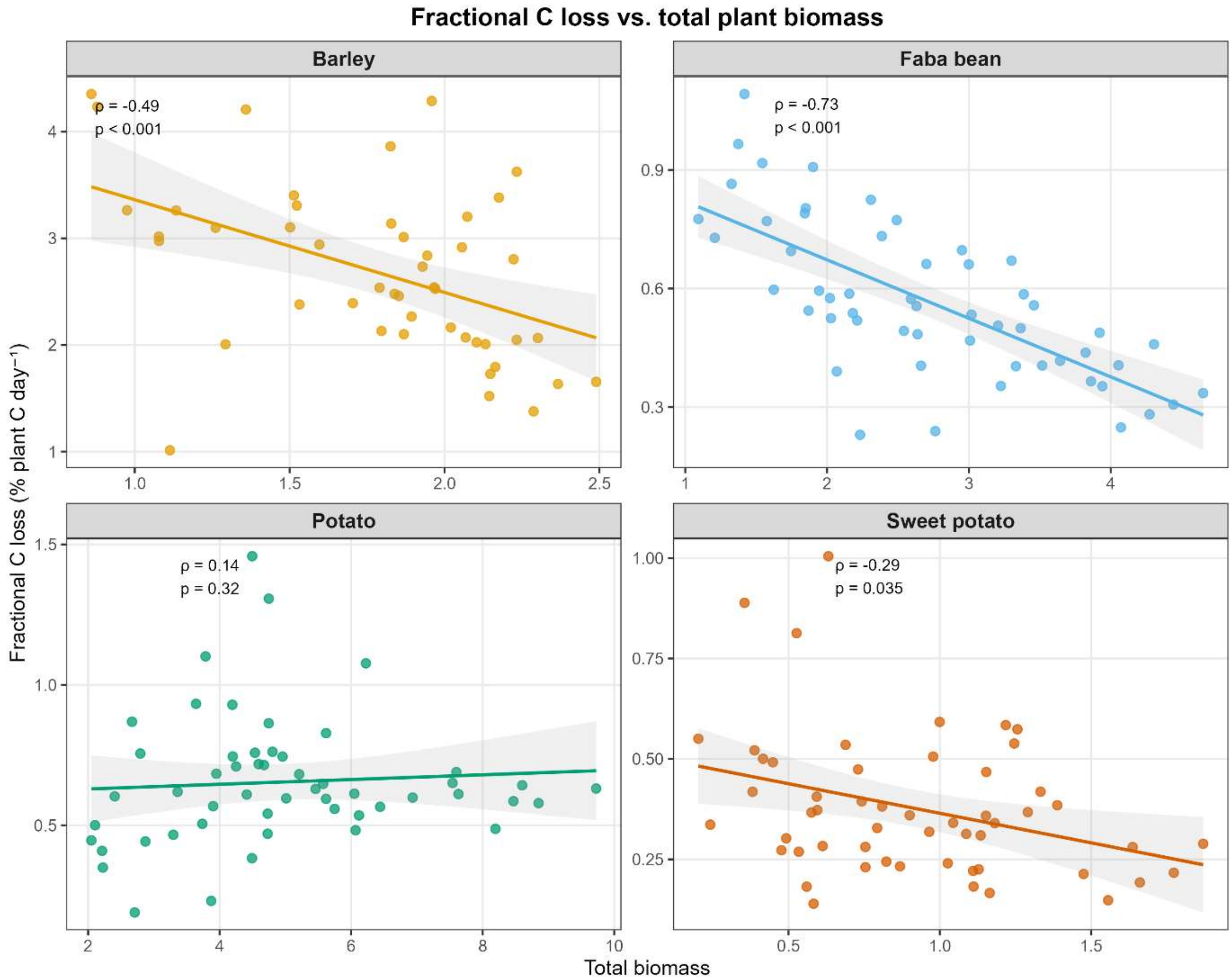

SI Fig. 5 Spearman rank correlation between fractional C loss (% plant C exuded day^{-1}) and total biomass (shoot + root dry weight in g) confirmed a significant negative relationship in three of four crops: Barley (ρ = −0.49, p < 0.001), Faba bean (ρ = −0.73, p < 0.001), and Sweet potato (ρ = −0.29, p = 0.035). Potato showed no significant relationship (ρ = 0.14, p = 0.320). Larger plants therefore release a smaller fraction of their C through root exudation, quantitatively supporting the negative correlation detected between plant biomass and root exudation rates per unit root surface area.

References

Badri DV, Vivanco JM (2009). Regulation and function of root exudates. Plant Cell Environ. 32. https://doi.org/10.1111/J.1365-3040.2008.01926.X

Dilkes NB, Jones DL, Farrar J (2004). Temporal Dynamics of Carbon Partitioning and Rhizodeposition in Wheat. Plant Physiol. 134:706. https://doi.org/10.1104/PP.103.032045

Jones DL, Nguyen C, Finlay RD (2009). Carbon flow in the rhizosphere: carbon trading at the soil–root interface. Plant Soil 321:5–33. https://doi.org/10.1007/s11104-009-9925-0

Ma S, He F, Tian D, Zou D, Yan Z, Yang Y, Zhou T, Huang K, Shen H, Fang J (2018). Variations and determinants of carbon content in plants: A global synthesis. Biogeosciences, 15:693–702. https://doi.org/10.5194/BG-15-693-2018
